# *LamelODF*: a MATLAB-based toolbox for orientation distribution analysis and mapping of lamellar minerals for laboratory and synchrotron X-ray diffractometers

**DOI:** 10.1107/S1600576726000968

**Published:** 2026-03-08

**Authors:** Baptiste Dazas, Eric Ferrage, Fabien Hubert, Brian Gregoire, Pierre Fertey, Laurent Michot

**Affiliations:** a Institut de Chimie des Milieux et Matériaux de Poitiers (IC2MP) Université de Poitiers CNRS PoitiersF-86073 France; b Synchrotron SOLEIL L’Orme des Merisiers, Départementale 128 Saint-Aubin91192 France; c Phenix, CNRS-UPMC-Sorbonne Université UMR 8234, 4 place Jussieu case courrier 51, Paris75005, France; University of Silesia in Katowice, Poland

**Keywords:** orientation distribution functions, 2D X-ray diffraction, lamellar minerals, MATLAB-based software, clay texture analysis, mapping

## Abstract

We present *LamelODF*, a MATLAB-based software platform for automated extraction and mapping of axisymmetric orientation distribution functions (ODFs) from 2D X-ray diffraction patterns of lamellar minerals. Building on a maximum-entropy-method-derived ODF specifically for clay mineral systems, the software provides a streamlined workflow for texture analysis under the assumption of transverse isotropy. The program features dedicated file converters for both laboratory and synchrotron data formats, batch processing capabilities, and flexible background-correction algorithms. The analytical pipeline performs azimuthal intensity integration with background subtraction, followed by non-linear fitting to extract quantitative orientation parameters including the 〈*P*_2_〉 order parameter, the deviation angle δ between the main orientation of lamellar particles and the detector reference, and integrated intensities, focusing specifically on basal 001 reflections to enable rapid processing of thousands of diffraction patterns for spatial mapping applications. Mapping of a laboratory-prepared porous clay medium and a natural soil sample demonstrates the software’s ability to detect density stratification, sedimentation discontinuities and complex geological structures such as relict topsoil crusts. The software successfully discriminates between different clay mineral phases, providing a practical complementary tool for researchers investigating the organization of lamellar minerals in natural and engineered materials.

## Abstract 2

*LamelODF* is a MATLAB-based toolbox aimed at simplifying the automated extraction of orientation distribution functions from 2D X-ray diffraction data of lamellar minerals in transmission mode. It accommodates both laboratory and synchrotron data formats, providing an integrated pipeline from raw data to spatially resolved textural maps, enabling robust quantification of orientation parameters and mineral phase discrimination.

## 1. Introduction

Preferred orientation of clay minerals and other lamellar phases plays a central role in controlling the physical properties of clay-rich materials, including permeability, diffusion and elastic anisotropy (Voltolini *et al.*, 2009 ▸; Backeberg *et al.*, 2017 ▸). In sedimentary rocks and soils, such textural anisotropy is of particular importance for geophysical interpretation, seismic prospecting and the modeling of transport processes (Wenk & Vasin, 2017 ▸; Wenk *et al.*, 2010 ▸) and has therefore been the subject of extensive experimental and theoretical investigation over several decades. The quantitative description of crystallographic textures is commonly expressed through the orientation distribution function (ODF), which defines the statistical relationship between crystal orientations and a sample reference frame. The general formalism of three-dimensional ODFs was established in the 1960s by both Bunge and Roe (Bunge, 1965 ▸; Roe, 1965 ▸), who independently developed methods for deriving crystallite orientation distributions from pole-figure data using expansions in spherical harmonics and generalized spherical harmonics, respectively. This approach has since been widely applied to a broad range of polycrystalline materials (Schaeben, 1988 ▸). For clay minerals and shales, full ODFs have been measured using synchrotron-based diffraction techniques for more than two decades (Wenk & Vasin, 2017 ▸; Wenk *et al.*, 2010 ▸; Voltolini *et al.*, 2009 ▸), often in combination with advanced analysis frameworks such as *MAUD* (Lutterotti *et al.*, 1999 ▸, 2010 ▸) and *MTEX* (Hielscher & Schaeben, 2008 ▸). These programs represent the most advanced numerical tools for texture analysis, enabling the refinement of 3D ODFs alongside phase fractions, particle sizes, and complex structural features such as turbostratic disorder in smectites or polytype intergrowths (Lutterotti *et al.*, 2010 ▸).

In many clay-rich systems, however, the crystallographic and morphological characteristics of phyllosilicates justify a potentially simplified description of texture. The strong platy habit of clay particles and the dominance of basal 00*l* reflections lead to a texture that can be reasonably approximated as transversely isotropic (Wenk *et al.*, 2010 ▸). Under this assumption, the three-dimensional orientation space reduces to a single angular variable describing the inclination of particle normals relative to a reference direction. This one-dimensional orientation distribution function, simply referred to as ODF in this study, is derived directly from the azimuthal intensity variation of a single 00*l* diffraction ring, rather than being reconstructed from multiple pole figures as in conventional texture analysis. Such axisymmetric orientation distributions have long been described using expansions in Legendre polynomials or spherical harmonics (Järvinen, 1993 ▸). A specific formalism embedding these mathematical expansions in exponential terms allows for a description based on the maximum entropy method (MEM). Early work, applied to uniaxial orientation distributions by Bower (1981 ▸), showed that maximizing the entropy under constraints of measured Legendre polynomial averages yields to an exponential distribution. This entropic approach was subsequently generalized to full three-dimensional texture goniometry by Schaeben (1988 ▸), who demonstrated that the MEM provides a mathematically rigorous and physically consistent way to reconstruct orientation distributions from incomplete experimental information. These concepts have since been applied and further developed in a variety of texture-analysis contexts, including reliability assessment of ODF refinements (Chateigner, 2005 ▸). More recently, Dabat *et al.* (2019 ▸) pro­posed a MEM-derived ODF specifically parameterized for lamellar systems, calibrated using experimental clay-rich samples and expressed as an exponential function of coupled Legendre polynomials. This formulation provides a compact and robust description of axisymmetric orientation distributions and is particularly well suited to clay minerals.

In parallel with these methodological developments, advances in X-ray diffraction instrumentation, most notably the widespread adoption (Donath *et al.*, 2023 ▸; Brönnimann & Trüb, 2018 ▸; Förster *et al.*, 2019 ▸) of hybrid photon-counting detectors (Taguchi *et al.*, 2008 ▸) and the increased brilliance of modern synchrotron sources (Bilderback *et al.*, 2005 ▸; Shin, 2021 ▸; Li *et al.*, 2022 ▸), have enabled the routine acquisition of large volumes of 2D X-ray diffraction (2D-XRD) data. Such datasets contain not only conventional structural information but also detailed azimuthal intensity variations that directly reflect preferred orientation. The main challenge has therefore shifted from data acquisition to data processing, especially in mapping experiments involving thousands of diffraction patterns for complex natural clay-rich materials (Geoffroy *et al.*, 2022*b* ▸; Manté *et al.*, 2020 ▸). In such cases the primary objective is not the reconstruction of a full three-dimensional ODF but rather the quantitative mapping of basal-plane alignment and its spatial variability across large heterogeneous samples prepared for transmission geometry, as demonstrated in a soil crust formation study (Geoffroy *et al.*, 2022*a* ▸). In this context, we present *LamelODF*, a MATLAB-based program designed for the automated extraction, fitting and mapping of axisymmetric ODFs of lamellar minerals from 2D-XRD data acquired on laboratory or synchrotron instruments. *LamelODF* builds explicitly on established theoretical foundations, particularly the MEM-derived ODF (Dabat *et al.*, 2019 ▸) for clay mineral systems. The program contribution lies in the integration of existing concepts into a workflow specifically designed for processing of transmission 2D-XRD data, where thousands of patterns must be analyzed to map a single orientation parameter in the millimetre to centimetre range. The software thus focuses on basal reflections, and, under the assumption of transverse isotropy, provides quantitative orientation parameters (order parameter 〈*P*_2_〉 and angular offsets) together with spatially resolved maps that are directly interpretable in geological and materials science contexts. This program thus complements existing comprehensive texture-analysis frameworks and provides a simple practical solution for researchers investigating the organization of lamellar minerals in natural and engineered materials.

## 2. Materials and data/methods

### 2.1. Mathematical derivation of the orientation distribution function

Dabat *et al.* (2019 ▸) developed a parametrized MEM-derived ODF specifically for clay minerals (CM), calibrated against six experimental samples spanning a wide range of anisotropic features:
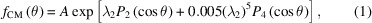
with, as in the classical MEM formalism, *A* the normalization constant, λ_2_ the Lagrange multiplier, and 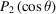 and 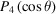 the second and fourth Legendre polynomials,
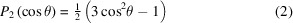
and
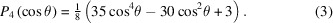
The empirical coupling coefficient (0.005) between λ_2_ and the fourth-order term constitutes the key element of this parametrization, rather than treating *P*_2_ and *P*_4_ as independent parameters. This experimentally derived constraint thus reduces the orientation description to a single adjustable parameter (λ_2_). Furthermore, truncation at the fourth order proves sufficient, as experimental validation demonstrates excellent agreement across the full range of orientation states encountered in natural and synthetic clay systems, with no need for higher-order terms.

Expanding expression (1) by substituting the Legendre polynomials (2) and (3) leads to the equation used for ODF determination in the software:
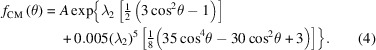
This expression provides a complete description of clay mineral orientation distributions with only three adjustable parameters: λ_2_, *A* and the angle θ. The latter is related to the detector reference angle θ_ref_ by θ = θ_ref_ − δ, where δ denotes the deviation angle between the main orientation of lamellar particles and the detector reference.

Finally, 〈*P*_2_〉 is classically defined as the ensemble average of 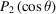 weighted by the ODF. It could thus be calculated with
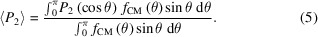
This parametrization provides a direct method for quantifying preferred orientation in lamellar materials, even from partially incomplete azimuthal data. The limited number of fitting parameters ensures robust convergence and fast computation, making it also suited for automated batch processing of the large datasets typically generated in mapping experiments.

### 2.2. Sample preparation protocol

**Figure 1:**
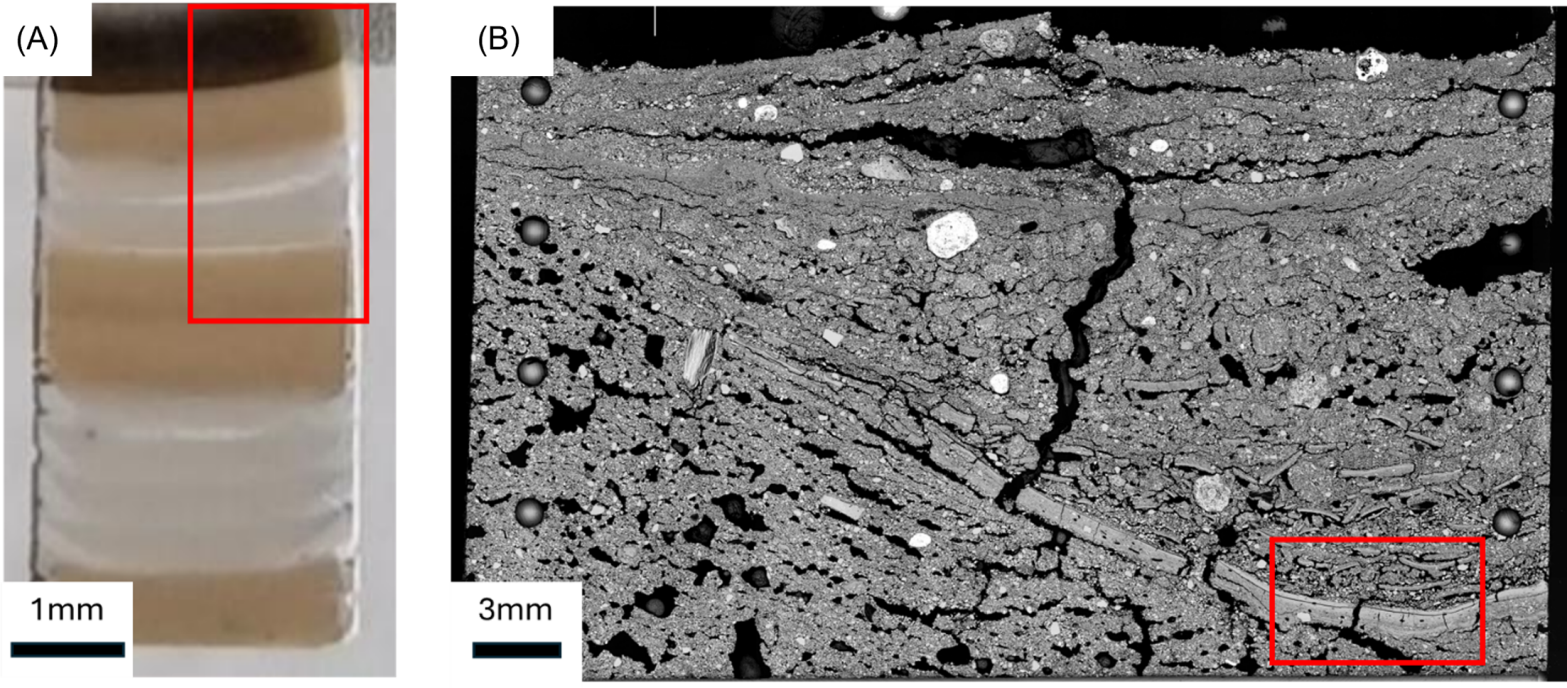
(*A*) Laboratory-prepared clay porous medium thin section after PMMA induration, composed of alternating beds of vermiculite (dark brown), mica (light brown) and kaolinite (white). The red area indicates the portion of the sample analyzed by 2D-XRD. (*B*) Scanning electron microscope image in secondary electron mode of the topsoil surface thin section of the control soil from the bare fallow experiment at INRAE Versailles. The red area indicates the portion of the sample analyzed by 2D-XRD.

Deriving a representative ODF from a transversely isotropic material requires careful sample preparation to ensure that the acquired data can be accurately and meaningfully interpreted within the framework defined above. In addition to transverse isotropy, optimal orientation of the sample with respect to the incident beam is essential: 2D transmission diffraction patterns must be collected perpendicular to the orientation plane to capture the full azimuthal intensity distribution of basal reflections. To illustrate both the analytical capabilities of *LamelODF* and the level of preparation required, we present below detailed protocols applied to two contrasting sample types: a laboratory-prepared synthetic clay medium with controlled texture [Fig. 1 ▸(*A*)] and a natural soil sample with complex depositional history [Fig. 1 ▸(*B*)]. The laboratory-prepared clay porous medium is composed of alternate beds of size-fractionated minerals KGa-2 kaolinite, from the Source Clays Repository of The Clay Mineral Society (CMS) at 0.5 µm, Santa Olalla vermiculite at 0.1–0.2 µm (Reinholdt *et al.*, 2013 ▸), and Poitiers University collection muscovite at <1 and 0.1–0.2 µm. The second sample is a preserved topsoil surface from a reference plot of the 42-plot long-term bare fallow experiment of INRAE Versailles (van Oort *et al.*, 2022 ▸).

#### 2.2.1. Centrifugation-induced laboratory-prepared clay particle orientation

The oriented clay porous medium sample used in this study was previously characterized and prepared (Dabat *et al.*, 2019 ▸) through a centrifugation protocol enabling the creation of centimetre-scale samples with controlled preferential particle orientation. For completeness, the preparation protocol involved sodium saturation of each selected clay mineral through three successive treatments with 1*M* NaCl solution, followed by dialysis until chloride ions were undetectable via silver nitrate testing, to ensure proper dispersion. After drying at 60°C and sieving through 50 or 150 µm mesh to control aggregate size, the powder was re-dispersed in ultrapure water using ultrasonic treatment, with suspension concentrations ranging from 2.5 to 50 g L^−1^ depending on desired depositional characteristics. The suspension was processed in specialized polytetrafluoroethylene (PTFE) tubes (6.4 mm internal diameter) using an Avanti J 301 centrifuge with a JS-24.38 rotor, configured for horizontal sedimentation to ensure particle deposition perpendicular to the tube walls.

The centrifugation parameters are modulated between 5000 r min^−1^ (4508*g*) and 10000 r min^−1^ (18032*g*) to control the deposition rate. The process involves successive cycles of centrifugation with 1–2 ml suspension aliquots, followed by careful removal of the supernatant after each cycle. This layered approach enables the creation of stratified samples, with the ability to incorporate multiple different clay types or deposition conditions within a single 1–2 cm sample [Fig. 1 ▸(*A*)]. Following centrifugation, the samples undergo a controlled drying phase at 50°C.

#### 2.2.2. Sample consolidation and sectioning

The preservation of the porous medium as well as the soil sample structure was achieved through resin impregnation using methyl methacrylate (MMA; C_5_H_8_O_2_), selected for its superior fluidity compared with water and its ability to rapidly infiltrate porous networks (Sammaljärvi *et al.*, 2012 ▸; Prêt *et al.*, 2004 ▸). Two distinct protocols were developed to accommodate the different sample characteristics and experimental requirements.

For the porous medium, housed in PTFE tubes (6.4 mm internal diameter), the impregnation process began with a brief vacuum treatment (2–5 min) to remove residual moisture. A mixture of MMA containing 0.5 wt% benzoyl peroxide (BPO) initiator was then introduced under vacuum conditions and maintained for 30 min to facilitate both liquid and vapor phase penetration. The impregnation period varied from three days to two weeks, depending on sample permeability. Polymerization was achieved through thermal initiation at 55°C for 24 h, resulting in conversion to polymethyl methacrylate (PMMA).

The soil samples, maintained in their collection cylinders and transferred to 160 ml polypropyl­ene containers, underwent a more gradual impregnation process due to their larger volume and complex pore structure. Following initial drying at 40°C for 24 h, samples were subjected to a 40 min vacuum treatment with a cold trap to remove residual capillary water. The MMA mixture, prepared with 0.27 wt% BPO, was introduced under vacuum and maintained for 45 min to ensure thorough pore infiltration. The impregnation period extended from one to two weeks, with longer durations applied to less permeable samples. Polymerization was conducted through a stepped temperature program (40, 45 and 50°C, each maintained for 48 h) to prevent rapid exothermic reactions in the larger sample volume. Both protocols resulted in well preserved porous structures suitable for subsequent analytical procedures, with the specific approach tailored to the unique characteristics of each sample type. The density change from liquid MMA (0.93 g cm^−3^) to solid PMMA (1.18 g cm^−3^) was accounted for in the initial resin volume calculations to ensure complete sample impregnation.

A critical challenge in preparing samples for transmission experiments lies in achieving the optimal balance between specimen thickness and signal quality. The sample thickness must allow adequate X-ray transmission while maintaining sufficient depth to ensure the diffracting volume contains a statistically representative particle population. For optimal data collection, two-dimensional transmission diffraction patterns must be acquired perpendicular to the sedimentation planes of the transversely isotropic mineral. Additionally, patterns can be recorded within the sedimentation planes, especially to verify the transverse isotropy of the material.

The laboratory-prepared porous medium presented here was vertically sectioned through its center using a water-cooled circular saw and then reduced to 0.5 mm thick slices using 15 µm pre-polishing paper. The slices were cut to dimensions of 6 × 12 mm, with the longer axis oriented parallel to the sample surface. For the natural samples (*e.g.* the soil sample), which typically present higher density, specimens were longitudinally sectioned using a small circular saw. The slab of 45 mm width × 30 mm height was carefully reduced to 1 mm thickness using finer 15 µm pre-polishing paper. Additional transverse sections were prepared in this protocol to verify the transverse isotropy of the clay porous media.

Sample characteristics significantly influence the preparation methodology for transmission analysis. While weakly cohesive samples require prior induration to maintain their structural integrity, more consolidated materials can be directly thinned to achieve optimal transmission thickness. Each preparation step is crucial for obtaining high-quality diffraction data. Although synchrotron facilities are commonly used for such acquisitions due to their superior beam characteristics, laboratory-based experimental setups can also provide valuable data. The choice between these experimental configurations depends on the specific requirements of the analysis, such as spatial resolution, acquisition time and beam energy.

### 2.3. Acquisition setup

**Figure 2:**
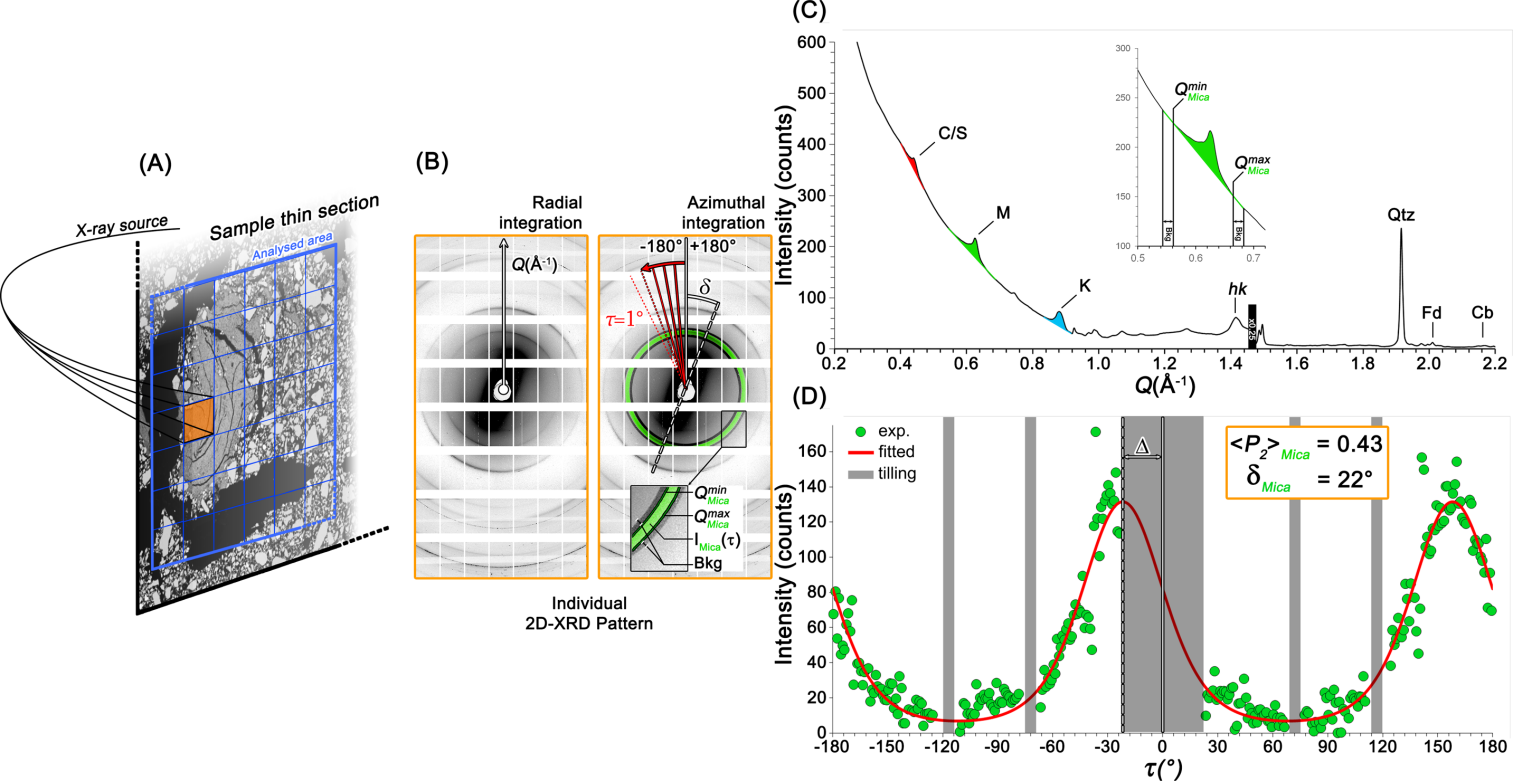
Experimental procedure allowing the computation of textural information for a natural clay system based on (*A*) sample mapping and acquisition on a properly prepared thin section with transmission 2D-XRD, generating (*B*) individual 2D-XRD patterns. Such patterns can be radially integrated along the **Q** vector, or azimuthally integrated along τ. Radial integration allows computation of a classical (*C*) 1D-XRD profile that allows the selection of 001 reflection peaks belonging to clay minerals [chlorite–smectite (C/S), mica (M) and kaolinite (K)]. Limits of peak integration (*Q*^min^ and *Q*^max^) can thus be defined, as well as background (bkg) width to individualize diffraction peaks from background. This selection is therefore repeated at every τ step to integrate signal and compute (*D*) the mineral orientation distribution, exemplified here in green for mica, where the parametrized MEM is fitted (red) to compute 〈*P*_2_〉 and δ. Geometrical detector features (tiles) appear here as gray areas on the pattern. Finally, computed parameters can be represented as a color-coded map as a function of their value.

Clay porous medium and soil sample data acquisition were performed at the CRISTAL beamline (https://www.synchrotron-soleil.fr/en/beamlines/cristal) of Synchrotron SOLEIL and are exemplified in Fig. 2 ▸. The transmission experimental setup utilized a monochromatic beam with an energy of 18.53 keV focused to a spot size of 76.85 µm. Thin samples were mounted on a goniometer head perpendicular to the incident beam and positioned on a motorized stage allowing translations along the *Y* and *Z* directions (with *X* aligned along the beam path) to facilitate sample mapping.

An XPAD3.2 X-ray pixel area detector was selected to maximize signal collection from potentially dilute mineral phases. This hybrid pixel array detector offers several advantages, including intrinsically low readout noise, high signal-to-noise ratio, rapid readout capabilities, extended dynamic range and excellent linearity (Le Bourlot *et al.*, 2012 ▸; Mocuta *et al.*, 2014 ▸). The detector’s architecture comprises 56 sensors arranged in eight modules across seven rows, with 3.5 mm dead zones between rows accommodating electronic connections. Each sensor features a 120 × 80 pixel array with 130 µm pixel size, except for the first and last horizontal columns, which are 2.5 times larger. This configuration yields a total active detection area of 153 × 75 mm (960 × 560 pixels).

The sample-to-detector distance was optimized to 375 mm to ensure the complete collection of clay mineral 00*l* diffraction rings. A lead beam stop centered on the detector provided protection from the direct beam. To prevent detector saturation from intense quartz reflections present in some samples, copper strips (35 µm thickness) were positioned at the upper and lower detector regions, attenuating high-intensity reflections while preserving the clay mineral 00*l* diffraction signals.

### 2.4. Data-treatment pipeline

**Figure 3:**
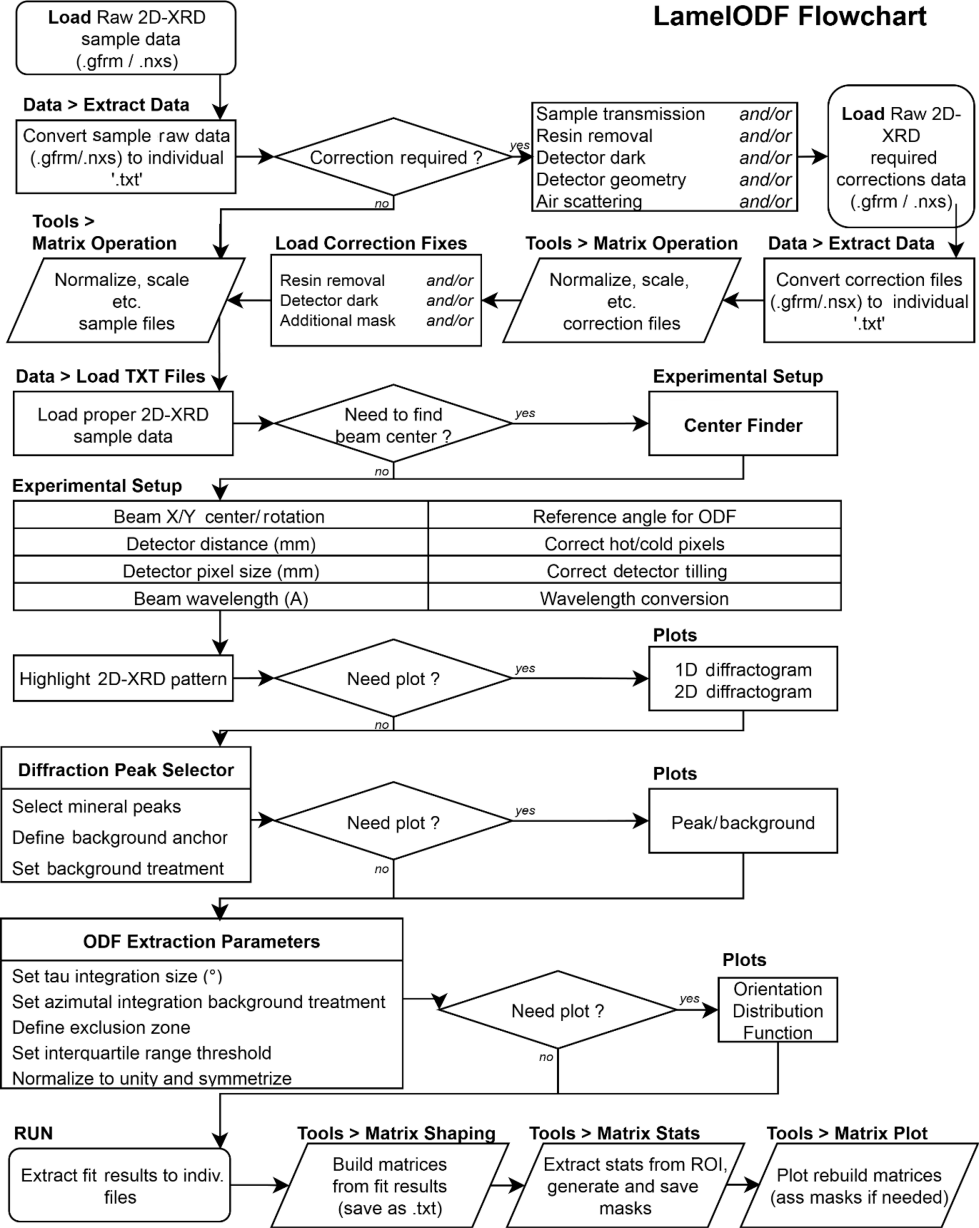
Workflow of the *LamelODF* software. The flowchart illustrates the main processing steps for orientation analysis from 2D-XRD data. Available functions are shown in bold, with key operations including data import, normalization, azimuthal integration, ODF reconstruction and parameter mapping. Decision points enable flexible workflows depending on the input type (single or batch processing) and analysis goals.

The analysis was implemented in MATLAB, a computational environment optimized for matrix operations that align well with two-dimensional image processing requirements. The developed script was designed with three key objectives: to accommodate diverse file formats and architectures from various 2D detectors, to handle variations in sample preparation and acquisition protocols, and to provide a flexible framework for ODF extraction and data mapping (Fig. 3 ▸ and Figs. SI.1–SI.3 of the supporting information).

#### 2.4.1. Raw data processing

2D-XRD often generates raw data in various formats, with NeXus files emerging as the internationally adopted standard for experimental data storage (Könnecke *et al.*, 2015 ▸; Klosowski *et al.*, 1997 ▸). However, the hierarchical architecture and data location within these files can vary significantly between instruments and facilities. Furthermore, alternative raw formats such as Bruker raw measurement files (.brml) containing embedded GADDS raw format (.gfrm) data also remain widely used in 2D-XRD. To address this heterogeneity, we adopted comma-separated text matrices (.txt) as the standardized format for data analysis in *LamelODF*. Any type of file converted to .txt matrices could therefore be loaded into the software, including widely used image export formats (.tiff, .png, .bmp *etc*.). To limit data conversion, we chose to implement file converters directly for raw data machine formats: .nxs and .gfrm. For NeXus files, the hierarchical tree structure can be inspected. Users can select multiple data paths simultaneously, enabling the extraction of different types of information (*e.g.* diffraction patterns, metadata) in a single operation. The converter implements batch processing capabilities, allowing multiple files with similar internal structures to be processed sequentially or via parallel computing when higher throughput is required. Data extraction preserves dimensionality: scalar values and matrices are extracted directly, while higher-dimensional data (3D or 4D) are interpreted as collections of 2D patterns, with each pattern saved as a separate file and named according to its position in the higher-dimensional space (*e.g.* scan number, *x*–*y* coordinates). This approach simplifies subsequent analysis by maintaining a consistent data structure regardless of the original file format or dimensional complexity. For laboratory diffractometer data, we implemented specialized routines to extract and process Bruker .gfrm files, addressing the specific format characteristics.

Following initial data extraction, a matrix calculation module enables efficient batch processing of datasets. This component supports essential mathematical operations (addition, subtraction, multiplication and division) that can be applied between individual files, between file lists or using scalar values. The implementation includes parallel processing capabilities to handle large datasets efficiently. This functionality facilitates tedious data-manipulation tasks, including background subtraction using reference patterns, normalization against monitor counts or exposure time, scaling to accommodate attenuation factors, and averaging across multiple patterns to improve signal-to-noise ratios.

#### 2.4.2. Geometric calibration and mask correction

**Figure 4:**
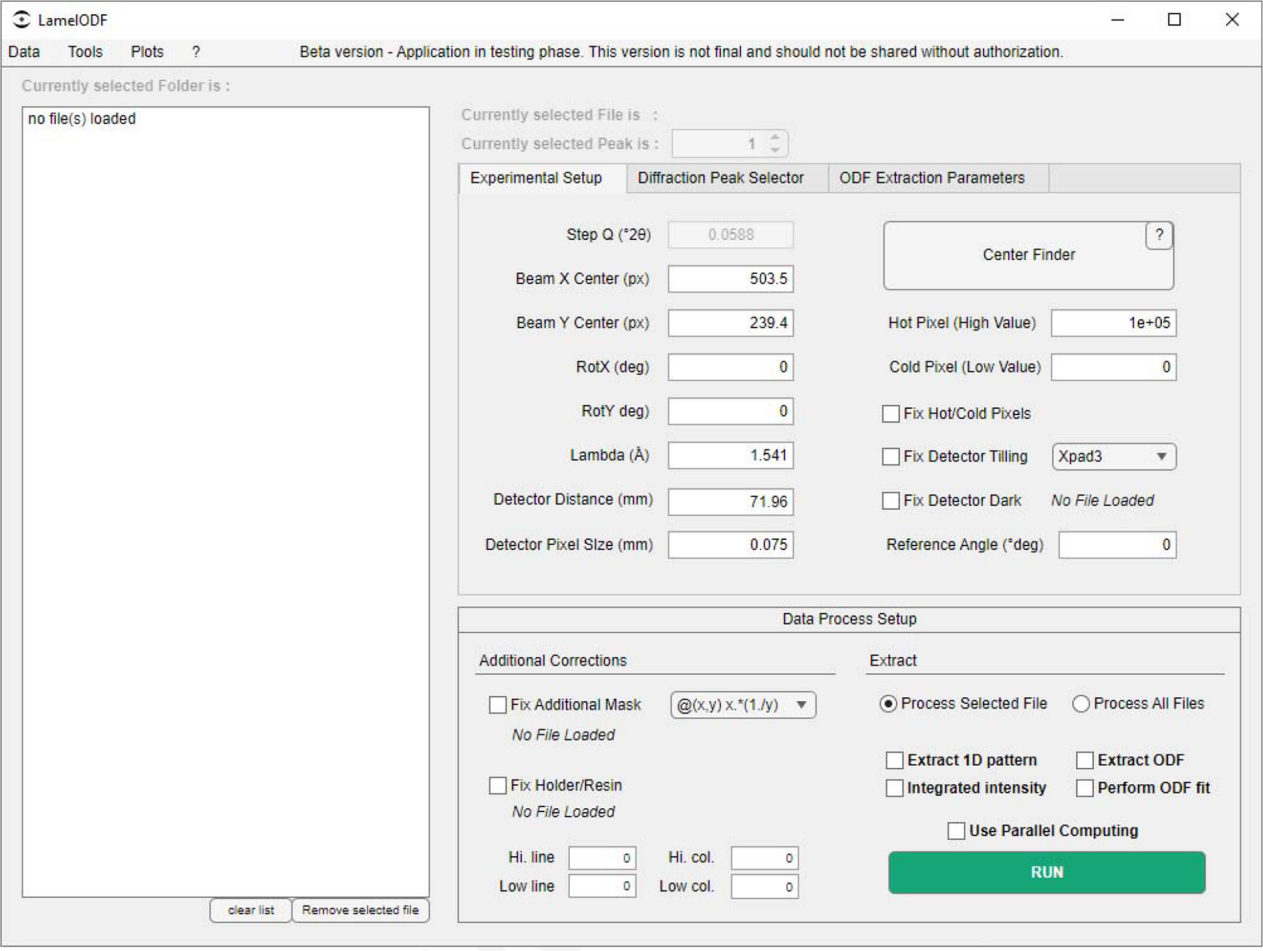
The *LamelODF* interface incorporates a banner that facilitates access to data loading and conversion, as well as backup capabilities. Various matrix operations and plots are available in the Tools section, while graphical features are located in the Plots section. The core parameters and setups are provided in the following sections: Experimental Setup, Diffraction Peak Selector and ODF Extraction Parameters. The Extract section is designed to facilitate the selection of extracted parameters and the execution of the script.

Once a properly formatted dataset is available, *LamelODF* (Figs. 4 ▸ and SI.1–SI.5) allows precise geometric calibration of the experimental setup. Our implementation employs a dual-stage optimization to determine critical geometric parameters. Initial calibration using the Center Finder function (Fig. SI.4) identifies the beam center through a semi-automated procedure where users select points along a diffraction ring. The algorithm employs minimization of the standard deviation of radial distances to determine the optimal center position that maximizes circular symmetry. Fine calibration through the Center Enhancer function provides an interactive interface with concentric overlay circles for precise adjustment of beam-center coordinates (*x*_0_, *y*_0_) and detector tilt angles around the *X* and *Y* axes. Finally, the user must specify the sample-to-detector distance and acquisition energy, here expressed as a wavelength in ångström units. The calibrated geometric parameters establish the mathematical framework for converting pixel coordinates to scattering angles [expressed in *Q* (Å^−1^) or converted into ° 2θ_B_ for any user-specified wavelength, with θ_B_ referring specifically to the Bragg angle to avoid confusion with the θ associated with ODFs] and azimuthal positions (τ), which are essential for subsequent integration operations.

The software includes additional correction mechanisms to address common experimental artefacts and sample-specific challenges. The Fix Additional Mask (Figs. 4 ▸ and SI.1) option allows users to apply a custom masking technique by performing mathematical operations between the dataset and a separate mask. Supported operations include multiplication (*xy*), division (*x*/*y*) and more, where *x* represents the original data and *y* represents the mask, both requiring matching matrix dimensions. This flexible approach enables precise data correction, tailored to the unique characteristics of individual experimental setups and detector configurations. The Fix Holder/Resin (Figs. 4 ▸ and SI.1) option provides a background-correction technique designed to remove systematic signal contributions from sample mounting materials or embedding resins. Users first load a reference file, typically the 2D-XRD signal from a pure sample holder or background material, and define a region of interest (ROI) within the 2D diffraction pattern. Then, the software compares the dataset with the background mask, computing the mean ratio between the two within the predefined ROI, thus requiring matching matrix sizes between files. This calculated background factor, denoted as factorB, is subsequently used to scale and subtract the background matrix from the original data, effectively eliminating unwanted signal contributions from the mounting medium or resin. Finally, during batch processing, the factorB value is automatically saved in individual files, ensuring precise tracking and documentation of the correction process.

#### 2.4.3. One-dimensional pattern generation

The one-dimensional pattern generator implements an optimized algorithm for radial integration of 2D diffraction patterns (Figs. 2 ▸, 5 ▸ and SI.4). The present implementation utilizes vectorized operations to significantly enhance computational efficiency. The algorithm proceeds by calculating the two-dimensional array of scattering angles (*Q*) for each pixel based on the calibrated geometry. It then defines angular bins based on an optimized stepsize equivalent to one pixel and pre-computes bin indices for all pixels in a single vectorized operation. The process continues by applying masks for valid data points (excluding NaN values and detector gaps), employing MATLAB’s accumarray function to efficiently compute both summed intensities and pixel counts for each angular bin, and finally calculating mean intensities by dividing summed values by corresponding pixel counts.

**Figure 5:**
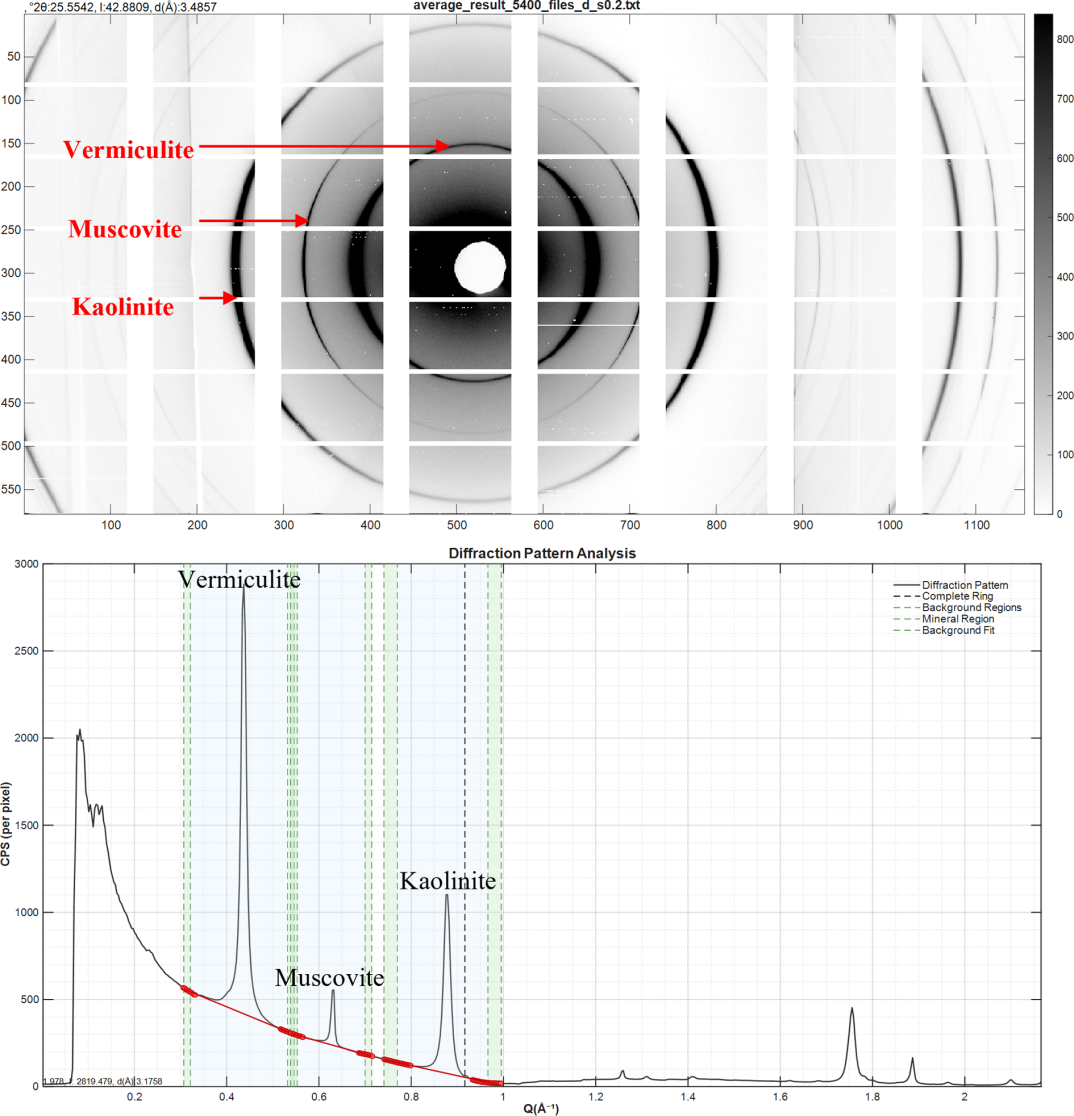
Software screenshots. The top image presents the 2D-XRD plots with ‘turbo’ colorbar, which are derived from the average of 5400 patterns of the sample of porous media, following proper calibration. As illustrated, the three primary rings are indicative of the three clay minerals’ 001 reflections. The grid observed in the data is caused by the geometric characteristics inherent in the XPAD3.2 detector design. The bottom image presents the corresponding radial integration of the top 2D-XRD pattern converted to copper radiation. The selection tool is employed to select the three peaks for subsequent azimuthal integration. As illustrated from left to right, the peaks correspond to the 001 reflections of vermiculite, muscovite and kaolinite. The background, later used to compute the integrated peak intensity, is displayed in red, while the background portions designated for azimuthal integration are shown in green. The dashed vertical black line indicates the threshold at which the rings are subjected to cropping.

The software provides an interactive interface through the Select Diffraction Peak function for selecting diffraction peaks of interest and defining corresponding background regions (Figs. 5 ▸ and SI.6–SI.15). This critical step establishes the angular ranges for subsequent orientation analysis or peak integration. Users select the *Q* range encompassing the diffraction peak of interest, two adjacent background regions (one on each side of the peak) and the azimuthal integration parameters including step size. The background regions are utilized for establishing baseline correction during azimuthal and intensity integration, ensuring accurate isolation of the diffraction signal. Transforming pathological pixels into NaN values allows subsequent statistical analyses to be performed while automatically excluding these data points from the computations.

#### 2.4.4. Azimuthal integration with background correction

**Figure 6:**
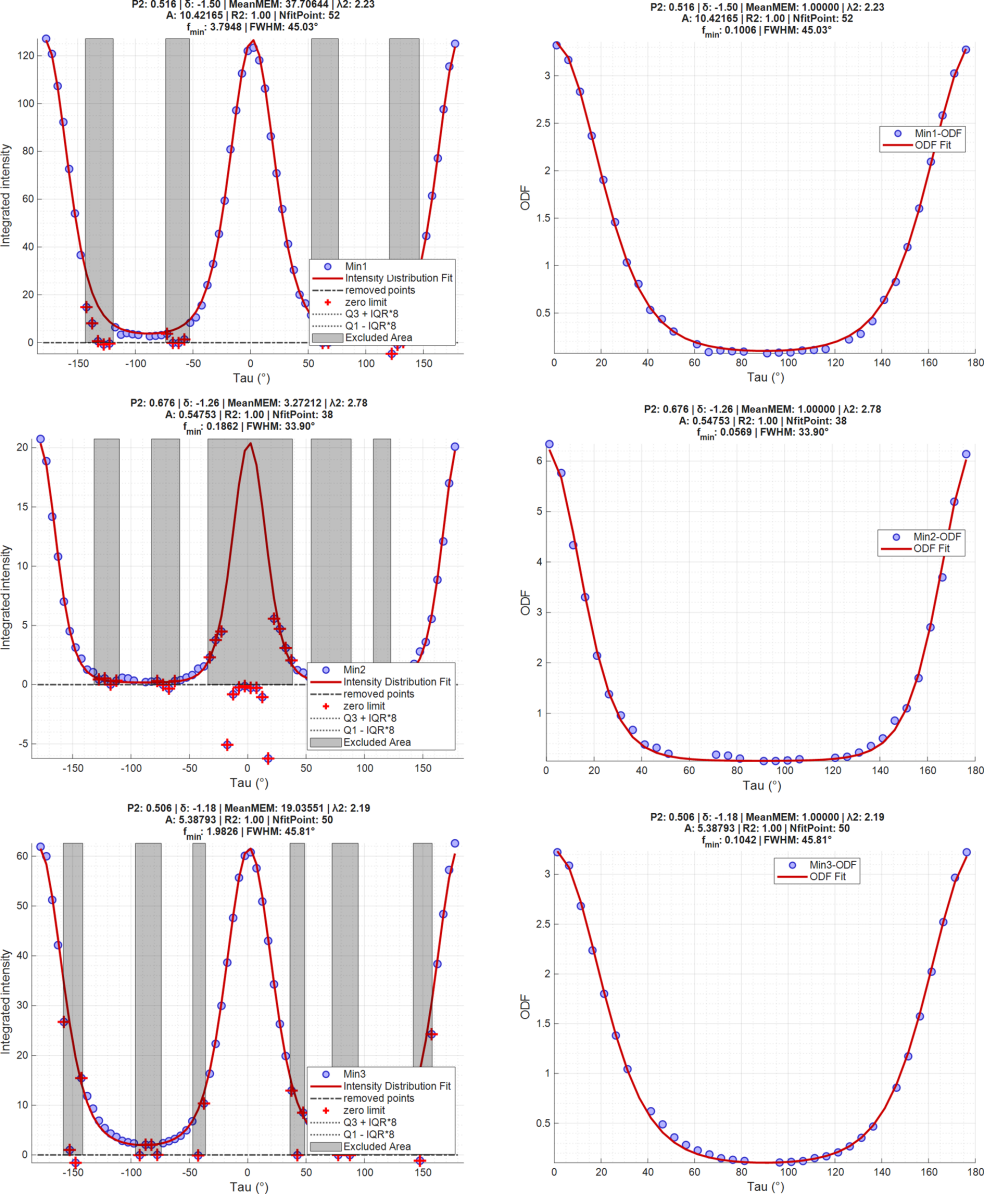
Screenshots of the ODFs derived from Fig. 5 ▸, with results arranged from top to bottom for vermiculite (min1), muscovite (min2) and kaolinite (min3). The left column shows the intensity distribution generated from azimuthal integration along τ; the right column shows the same data expressed as a symmetrized and normalized ODF with removal of excluded data. Blue dots represent experimental data, while the red line indicates the fitted ODF function. No peak-integration function was used in this case. Excluded data are denoted by red crosses. Data may be excluded either because they fall within a predefined exclusion area (manually delineated based on detector tiling) or because they lie outside the pre-established IQR. The plots are direct screenshots from the ODF Plot option, and the titles summarize fitting parameters, with 〈*P*_2_〉 = 0.516, 0.676 and 0.506 for vermiculite, muscovite and kaolinite, respectively.

The azimuthal integration function represents the core analytical component for ODF extraction. This algorithm isolates the orientation-dependent signal from the background, performing the integration in τ space (azimuthal angle around the diffraction ring). For each azimuthal position τ_*i*_ (from −180° to 180°), the algorithm identifies pixels within the angular sector τ_*i*_ to τ_*i*_ + Δτ (where Δτ is the user-defined azimuthal step). It then extracts intensity values for these pixels from both the peak region and the adjacent background regions (Figs. 2 ▸ and 5 ▸), constructs one-dimensional patterns as a function of *Q* for this specific azimuthal sector, and fits the background via the user-selected mathematical model (Figs. 5 ▸ and SI.1B): polynomial fitting with adjustable order (1–10), cubic spline interpolation for complex background shapes, piecewise cubic Hermite interpolating polynomial (PCHIP) for monotonic behavior and modified Akima piecewise cubic Hermite interpolation (makima) for reduced oscillations. The choice of background model depends on the specific characteristics of the diffraction data, with polynomial models typically providing robust performance for most clay mineral diffraction patterns. The process continues by subtracting the fitted background from the peak region and integrating the resulting background-corrected intensities to obtain the ODF value at position τ_*i*_. For the final integration step, the user can select from three methods (Fig. SI.1C): direct numerical integration (None option), Gaussian peak fitting or lognormal peak fitting. The None option is particularly advantageous for clay minerals, as it does not make assumptions about the distribution shape and allows for direct calculation of integrated intensity. While this direct integration approach is robust and ensures straightforward background correction when an appropriate background model is selected (Figs. 6 ▸ and SI.6–SI.15), ODF extraction becomes challenging in complex background conditions, particularly after extensive data preprocessing (*e.g.* background removal, resin removal). In such cases, accurate background estimation may be difficult to achieve with this method. Therefore, when background modeling is problematic, the Gaussian or lognormal fitting approaches are recommended, as they can effectively integrate the signal even when background subtraction results in potentially negative intensities.

#### 2.4.5. ODF fitting

The implementation employs a nonlinear least squares approach, optimizing three primary parameters: λ_2_ (the orientation parameter related to the degree of preferred orientation), δ (the angular offset parameter expressed in degrees that quantifies the azimuthal shift between the observed distribution and the theoretical reference position on the detector frame) and *A* (the amplitude factor related to diffraction intensity). The theoretical reference position for δ is defined, by default, as vertical, with 0° pointing directly upward in the image frame. Angles increase clockwise from 0° to +180° (when pointing directly downward). At the downward position, the value transitions from +180° to −180° and continues increasing clockwise from −180° back to 0° at the upward position. Users should be careful about their sample orientation when interpreting these values. The reference angle parameter allows for adjusting this theoretical reference position as needed for proper alignment. From the fitted ODF, the algorithm outputs multiple parameters (Figs. 6 ▸ and SI.6–SI.15): The 〈*P*_2_〉 value (second-order orientation parameter) is computed as detailed in equation (5). The λ_2_ parameter corresponds directly to the fitted λ_2_ value. The MeanMEM parameter represents the integrated value of the intensity distribution across all azimuthal angles. The NfitPoint parameter indicates the number of data points used in the fitting process after excluding outliers and user-defined exclusion zones, identified through quartile-based statistical methods and user-defined exclusion regions. The *f*_min_ parameter represents the minimal value of the fitted function, and the FWHM represents its full width at half-maximum. Furthermore, the *R*^2^ coefficient of determination quantifies the fitting quality, with values approaching 1 indicating excellent agreement between the experimental data and the fitted model. The symmetrized and normalized-to-unity ODF can be explicitly displayed by selecting the ‘Normalize to unity’ option (Figs. SI.1C, 6 ▸ and SI.6–SI.15), while the actual intensity distribution is displayed if this option is not turned on. This only affects the MeanMEM and *f*_min_ parameters in the displayed results.

The algorithm incorporates several features to enhance fitting robustness: adjustable threshold parameters for outlier detection, user-defined exclusion zones to account for experimental artefacts (detector gaps, beam stops), optimized initial parameter estimation based on peak position and intensity, and multiple fitting attempts with varied starting conditions if initial convergence fails. This comprehensive approach enables reliable analysis even for complex orientation distributions or datasets with limited angular coverage.

#### 2.4.6. Batch processing and visualization

**Figure 7:**
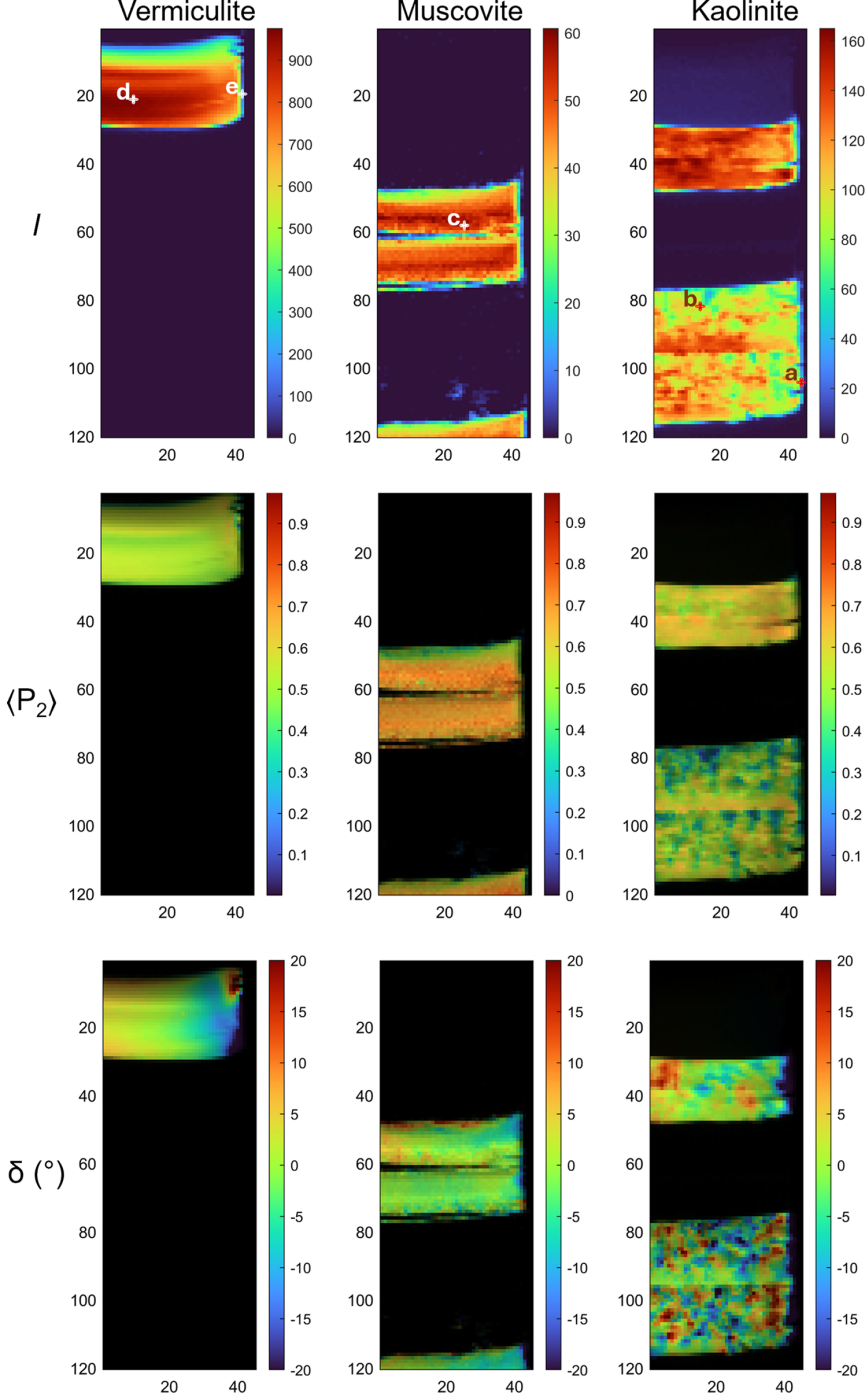
Screenshots of reconstructed maps using the Indiv. file To Matrix and Matrix Plot tools for the intensity (*I*), 〈*P*_2_〉 and δ parameters computed from the ODF fit. The minerals vermiculite, muscovite and kaolinite are selected on the basis of the three predefined zones shown in the average plot (Fig. 5 ▸) and applied in batch for every 2D-XRD file with the strategy illustrated in Fig. 2 ▸. The intensity of each mineral was used as a mask to represent 〈*P*_2_〉 and δ, thus preventing the appearance of abnormal values. For the pixels highlighted in the intensity plot and labeled a, b, c, d and e, the fitting strategy and results are detailed in Figs. SI.6–SI.10, respectively.

To accommodate large datasets from mapping experiments, we implemented a batch processing framework (Fig. SI.1). This component enables systematic analysis of multiple files with optional parallel computation to leverage multi-core architectures. The implementation thus allows for processing multiple mineral phases and multiple analytical procedures, including 1D pattern extraction, peak-intensity integration, ODF extraction and ODF fitting. Results are organized in a hierarchical directory structure that preserves the relationship between raw data and derived parameters, facilitating subsequent statistical analysis and visualization. For each mineral phase, separate subdirectories store the extracted 1D patterns, ODF data, fitted parameters and integrated intensities. Complex mappings can be entirely rebuilt, as the matrix shaping tool (Data Shaping) (Fig. SI.5) allows users to transform multiple one-dimensional text files into singular two-dimensional maps through an intuitive interface that enables dynamic data reorganization. To further extend the analytical capabilities, two additional tools are available, ROI to Histogram and Histogram to ROI, to post-process generated maps. The first enables the spatial analysis of maps by allowing definition of arbitrary polygonal ROIs directly on the visualized matrix. Users can thus statistically characterize selected regions through automatically generated histograms and quantitative metrics including mean, median and standard deviation. The second allows for selecting ranges of values directly on the map intensity histogram. This selection mechanism automatically highlights corresponding spatial regions on the map visualization, enabling rapid identification of structures with similar compositional or physical properties. Together, these tools bridge the gap between spatial and statistical analysis, allowing researchers to move seamlessly between visual patterns and their underlying quantitative distributions. Finally, the Matrix Plot (Figs. 7 ▸, 8 ▸ and SI.3) tool complements the previous tool by allowing researchers to load matrix data and apply visual transformations. Users can adjust color scaling, modify contrast levels and explore data through various color maps, enabling nuanced insights into complex datasets. The application supports multiple matrix operations, such as rotation, flipping and zooming, which facilitate detailed visual inspection. Critically, it preserves the original data integrity while offering interactive exploration, making it an essential tool for researchers seeking to extract visual meaning from numerical data.

## 3. Applications

### 3.1. Clay porous medium sample

Data were extracted from a single 11.4 GB .nxs file containing a 2D-XRD mapping of the clay porous medium sample. This map comprised 5400 diffraction points (120 × 45 grid) with a 0.2 s acquisition time per point, representing a total surface area of 30.375 mm^2^ [red rectangle in Fig. 1 ▸(*A*)]. Selected diffraction points are shown as examples in Figs. SI.6–SI.10. NeXus extraction tools were used to extract individual diffraction files. This extraction process interpreted the higher dimension of the NeXus file as sequential steps, generating 5400 individual .txt files. To standardize analysis, each diffraction file was normalized to a 1 s counting time using the Matrix Operation tool. Additionally, an average diffraction file was generated to facilitate the selection of all mineral peaks and subsequent center adjustments.

The average datafile (Fig. 5 ▸) was loaded into the main *LamelODF* interface for analysis. Beam centering was precisely conducted using the Center Finder tool, which determined beam coordinates at *X* = 521.4 pixels and *Y* = 288.5 pixels, with no rotational adjustment required. The detector distance was finely calibrated to 220.2 mm on the basis of the known *d*-spacing value for kaolinite. This calibration positioned the 001 peak reflections of kaolinite, mica and vermiculite at 7.15, 9.96 and 14.36 Å, respectively. The detector settings were optimized by establishing hot and cold pixel value limits at 1 × 10^5^ and 0, respectively. A detector dark acquisition was incorporated to account for background noise, and the detector’s specific architecture was accommodated by selecting the XPAD3 option. For comprehensive background correction, an acquisition scan of the pure resin binding the sample was extracted, averaged and normalized to 1 s and then applied using the Fix Holder/Resin option. The background-evaluation ROI was precisely defined from line 70 to 30 and column 630 to 600. The reference angle was set to +90° to account for the sample rotation.

The main 001 diffraction peaks of the three minerals were carefully selected to ensure centered peaks with sufficient width to completely encompass each diffraction signal. Selection zones were defined as 0.21, 0.15 and 0.04 Å^−1^ wide for vermiculite, muscovite and kaolinite, respectively. The background zone sizes were set to 0.015 Å^−1^ for accurate background evaluation, with the background width parameter maintained at 1. A first-order polynomial was employed to fit the background signal. ODF data were extracted with a step τ of 5° and an interquartile range (IQR) threshold of 8. Peak integration for the ODF was not required for this analysis and was therefore set to None. To compensate for anomalous datapoints caused by detector tiling artefacts, multiple exclusion zones were established for each mineral species (Fig. 4 ▸).

Following this setup, all 5400 datafiles were loaded and processed. The ODF, ODF fit, 1D diffraction patterns and peak integrated intensities were successfully extracted for the analysis of the clay mineral orientations throughout the sample. The mapping tool (Fig. SI.3) allows for reconstructing intensity, 〈*P*_2_〉 and τ maps, with an alpha mask based on the intensity maps also used for better representation of the latter two parameters (Figs. 7 ▸ and SI.3). Analysis reveals distinct sedimentation patterns across vermiculite, muscovite and kaolinite minerals. Vermiculite exhibits density stratification within single beds, with higher compaction and particle orientation at the bottom. Both muscovite and kaolinite clearly display structural discontinuities between sub-beds, with muscovite showing complete layer separation during drying. While muscovite sub-beds prepared under identical conditions demonstrate consistent structural properties, kaolinite’s behavior varies significantly with concentration. Higher concentrations (50 g L^−1^) lead to denser, more oriented, particles in the top bed, with less-oriented aggregates in the middle and bottom sub-beds prepared at higher gravitational fields. Muscovite, at lower concentrations (25 g L^−1^), remains relatively unaffected by gravitational-field variations. Additionally, all minerals show disturbed sedimentation patterns near container walls, characterized by decreased density and increased porosity. These findings demonstrate the technique’s sensitivity to sedimentation events, deposition conditions and topographical effects on clay mineral structuring.

### 3.2. Natural clay soil sample

Following a similar procedure as for the clay porous medium, the data were extracted from a single 10.2 GB .nxs file containing a 2D-XRD mapping of the soil top surface. This map comprised 4459 diffraction points (91 × 49 grid) with a 4 s acquisition time per point, representing a total surface area of 23.03 mm^2^ [red rectangle in Fig. 1 ▸(*B*)]. Selected diffraction points are shown as examples in Figs. SI.11–SI.15. NeXus extraction tools were used to extract individual diffraction files. To standardize analysis, each diffraction file was normalized to a 1 s counting time using the Matrix Operation tool. Additionally, an average diffraction file was generated to facilitate the selection of all mineral peaks and subsequent center adjustments.

The average datafile was loaded into the main *LamelODF* interface for analysis. Beam-centering coordinates were adjusted to *X* = 495 pixels and *Y* = 288 pixels, with no rotational adjustment required along either *X* or *Y* axes. The wavelength was set to 0.6709 Å with detector distance calibrated to 375 mm and a detector pixel size of 0.13 mm. The detector settings were maintained with hot and cold pixel value limits at 1 × 10^5^ and 0, respectively. The XPAD3 detector option was selected to accommodate the detector’s architecture. For background correction, a copper mask multiplied by *x*(1/*y*) was applied, and the resin-sample background-evaluation region was defined from pixel positions 30 to 70 lines and 600 to 630 columns. The reference angle was adjusted to −90° to account for sample rotation during acquisition.

The diffraction peaks of three minerals were selected with the following *Q* ranges: 0.3673–0.4844 Å^−1^ for smectite (S) and/or illite/smectite (IS) related diffraction peaks, 0.5620–0.6725 Å^−1^ for illite, and 0.8264–0.9137 Å^−1^ for kaolinite. ODF data were extracted with a step τ of 5° (same as previous) and an IQR threshold of 10. Peak integration for the ODF was set to ‘Gaussian integration’ for better integration of a less continuous signal (Fig. 4 ▸).

**Figure 8:**
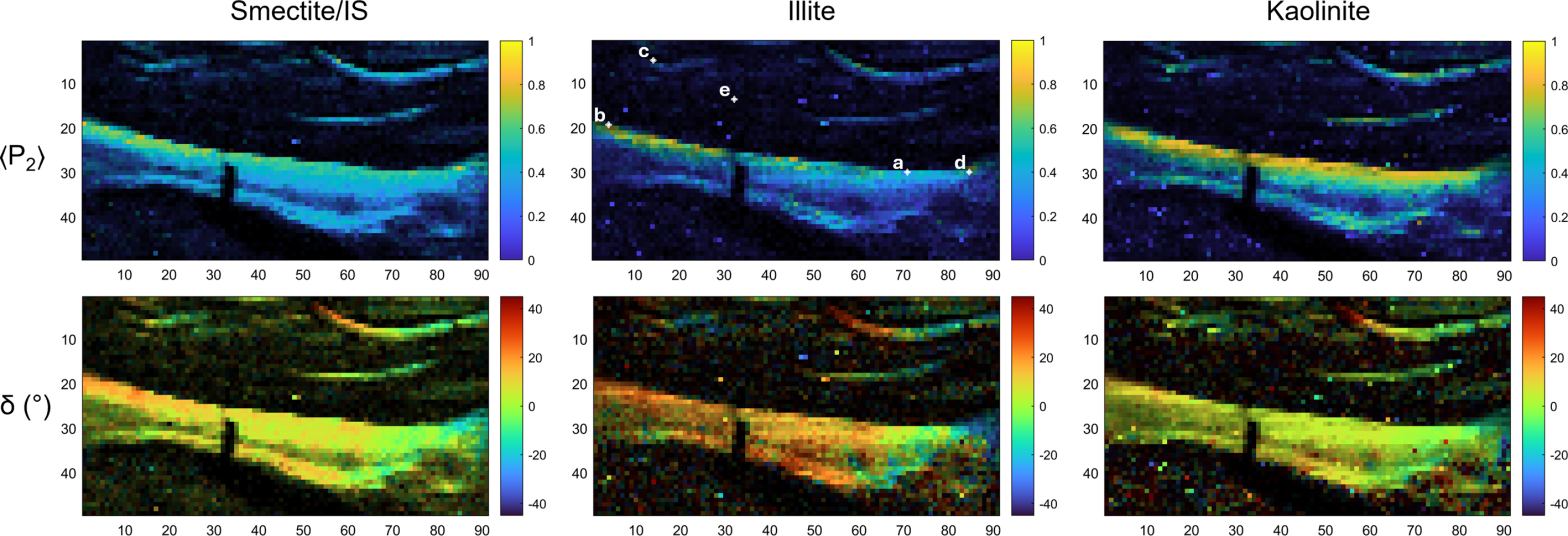
Reconstructed maps using the Indiv. file To Matrix and Matrix Plot tools for the 〈*P*_2_〉 and δ parameters computed from the ODF fit. The minerals smectite/IS (illite/smectite), illite and kaolinite are selected on the basis of the three predefined zones selected in the average plot and applied in batch for every 2D-XRD file. The intensity of each mineral was used as a mask to represent 〈*P*_2_〉 and δ, thus preventing the appearance of abnormal values. For the pixels highlighted in the illite 〈*P*_2_〉 plot and labeled a, b, c, d and e, the fitting strategy and results are detailed in Figs. SI.11–SI.15, respectively.

The parameter maps obtained from analysis illustrate distinctive structural organization across three clay mineral phases: S/IS, illite and kaolinite. The 〈*P*_2_〉 parameter maps (Fig. 8 ▸) clearly highlight contrasted mineral distributions, with all three minerals exhibiting pronounced layered architectures characterized by very high 〈*P*_2_〉 values (0.6–0.8), accompanied by moderate to high τ values (0.2–0.6). 〈*P*_2_〉 analysis reveals multiple relicts of former topsoil crusts (maximum ∼1.1 mm for the central thickest piece) that have been reworked and dislocated in this upper soil horizon. The distinctive formation of these soil structures, through surface sealing and particle sedimentation, generates fine but extremely oriented features clearly visible here. All three minerals are present within these structures, but they exhibit varying degrees of anisotropy. Comparable organization patterns observed in this natural sample mirror those seen in laboratory clay porous medium studies. The degree of anisotropy increases from bottom to top, reflecting a deposition process distinct from that in the clay porous medium, and likely more influenced by evaporation. One can clearly distinguish at least two different sedimentation events from the main central crust. Smaller crust structures are also visible, though their dimensions approach the beam size, making them only a couple of pixels thick at maximum. Despite insufficient resolution for observing stratification heterogeneity, anisotropy and orientation remain observable. Regarding anisotropy, kaolinite presents the highest 〈*P*_2_〉 values, followed by illite and S/IS with similar value ranges. The δ parameter is most pronounced for illite, which demonstrates the most significant orientation contrasts, while S/IS and kaolinite display rather similar orientational features. The remarkable similarity in structural patterns across all three mineralogical phases suggests comparable sedimentation behaviors despite their inherent crystallographic differences, while simultaneously revealing subtle variations in their response to depositional conditions.

## 4. Limitations and perspectives

Several limitations should be acknowledged when using the program for orientation analysis. This approach does not allow refinement of phase fractions, particle sizes and shapes, lattice parameters, or polytype distributions. The software is therefore not suited for applications requiring complete crystallographic texture characterization or the calculation of anisotropic physical properties from full ODFs. It is specifically designed for materials exhibiting transverse isotropy, where particle orientations are statistically axisymmetric around a single reference direction. This fundamental assumption, while valid for many sedimentary clay deposits and laboratory-prepared samples, may not hold for all geological materials, particularly those that have undergone tectonic deformation or metamorphism. Users must verify that samples meet this criterion before analysis, ideally by acquiring additional diffraction patterns within the sedimentation plane to confirm axial symmetry. Furthermore, 2D transmission diffraction data must be collected perpendicular to the main orientation plane to capture the full azimuthal intensity distribution of basal reflections. Failure to meet these geometric requirements will result in unreliable orientation parameters. The current implementation does not include peak-deconvolution capabilities. When multiple clay mineral phases contribute to overlapping 001 reflections, as commonly occurs with interstratified phases, the software cannot separate individual contributions. Users must therefore exercise careful judgment in peak selection, favoring well resolved reflections and adopting a parsimonious approach when dealing with complex mineral assemblages. This limitation is particularly relevant for poorly crystalline or turbostratically disordered clay minerals, which exhibit significantly broadened and asymmetric peaks that may conceal multiple contributions.

Several enhancements are envisaged to address current limitations and extend the software’s capabilities. These include (i) implementation of additional texture strength metrics such as texture index and texture entropy, to facilitate comparison with ODFs derived by other methods; (ii) standard error estimation on refined parameters; (iii) expanded file format support and improved cross-platform compatibility; and (iv) potential integration of basic peak-fitting routines for partially overlapping reflections.

## 5. Conclusions

The program *LamelODF* provides a practical solution for automated extraction and spatial mapping of ODFs from 2D-XRD data of lamellar minerals. The software builds explicitly on established theoretical foundations, particularly the MEM-derived ODF parameterized for clay mineral systems. The software architecture addresses key practical challenges in 2D-XRD data processing: it accommodates diverse file formats from both laboratory and synchrotron instruments through dedicated converters, handles large datasets through optimized batch processing with optional parallel computation, and provides flexible analytical workflows including multiple background-correction strategies. The focus on basal 00*l* reflections and the limited number of fitting parameters ensure robust convergence and fast computation, enabling the analysis of thousands of diffraction patterns required for millimetre- to centimetre-scale mapping experiments.

Validation through two contrasting sample types demonstrates the software’s reliability and practical utility. Analysis of the laboratory-prepared porous clay medium reveals sensitivity to subtle structural variations, including density stratification within individual beds, sedimentation discontinuities between layers and wall effects near container boundaries. The natural soil sample analysis showcases the capability to identify and characterize complex geological/pedological structures, including multiple relict topsoil crusts and distinct sedimentation events, providing quantitative insights into pedological processes. The extracted parameters, particularly 〈*P*_2_〉 and δ, enable direct comparison of structural organization across different clay mineral phases and depositional environments.

*LamelODF* addresses a specific requirement where spatial mapping of preferred orientation in transversely isotropic materials is the primary objective. This makes the software particularly suited for applications in soil science, sedimentology, and materials engineering where large-area textural characterization takes precedence over complete crystallographic texture determination.

## Supplementary Material

Additional figures. DOI: 10.1107/S1600576726000968/jur5011sup1.pdf

## Data Availability

The compiled Windows version of *LamelODF*, along with the full source code, is available at https://src.koda.cnrs.fr/baptiste.dazas.1/lamelodf.
