# Supplementary material for: LamelODF: a MATLAB-based toolbox for orientation distribution analysis and mapping of lamellar minerals for laboratory and synchrotron X-ray diffractometers
Source: J Appl Crystallogr. 2026 Mar 8;59(Pt 2):648–61. doi: 10.1107/S1600576726000968 (PMC13060612; doi:10.1107/S1600576726000968)
Supplement: Supplementary file 1 [file j-59-00648-sup1.pdf]

JOURNAL OF APPLIED CRYSTALLOGRAPHY

**Volume 59 (2026)**

**Supporting information for article:**

# *LamelODF*: a MATLAB-based toolbox for orientation distribution analysis and mapping of lamellar minerals for laboratory and synchrotron X-ray diffractometers

**Baptiste Dazas, Eric Ferrage, Fabien Hubert, Brian Gregoire, Pierre Fertey and Laurent Michot**

## SI.1 A.

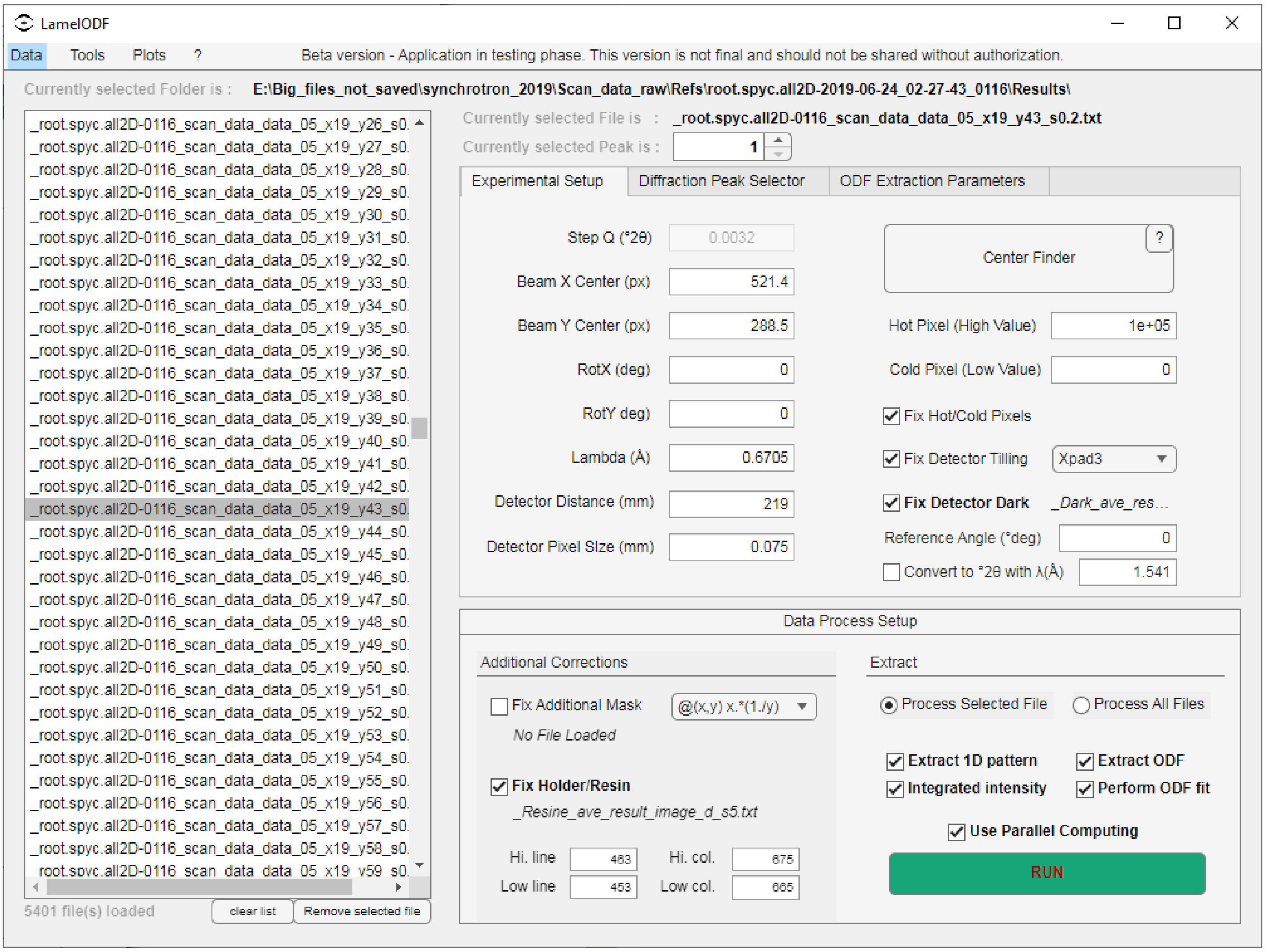


**SI.1:** A. Main lamelODF software window, with loaded example files, experimental setup, and data processing setup. B. is similar but with the diffraction peak selector option and C. display the ODF extraction parameters setup.

## SI.1 B.

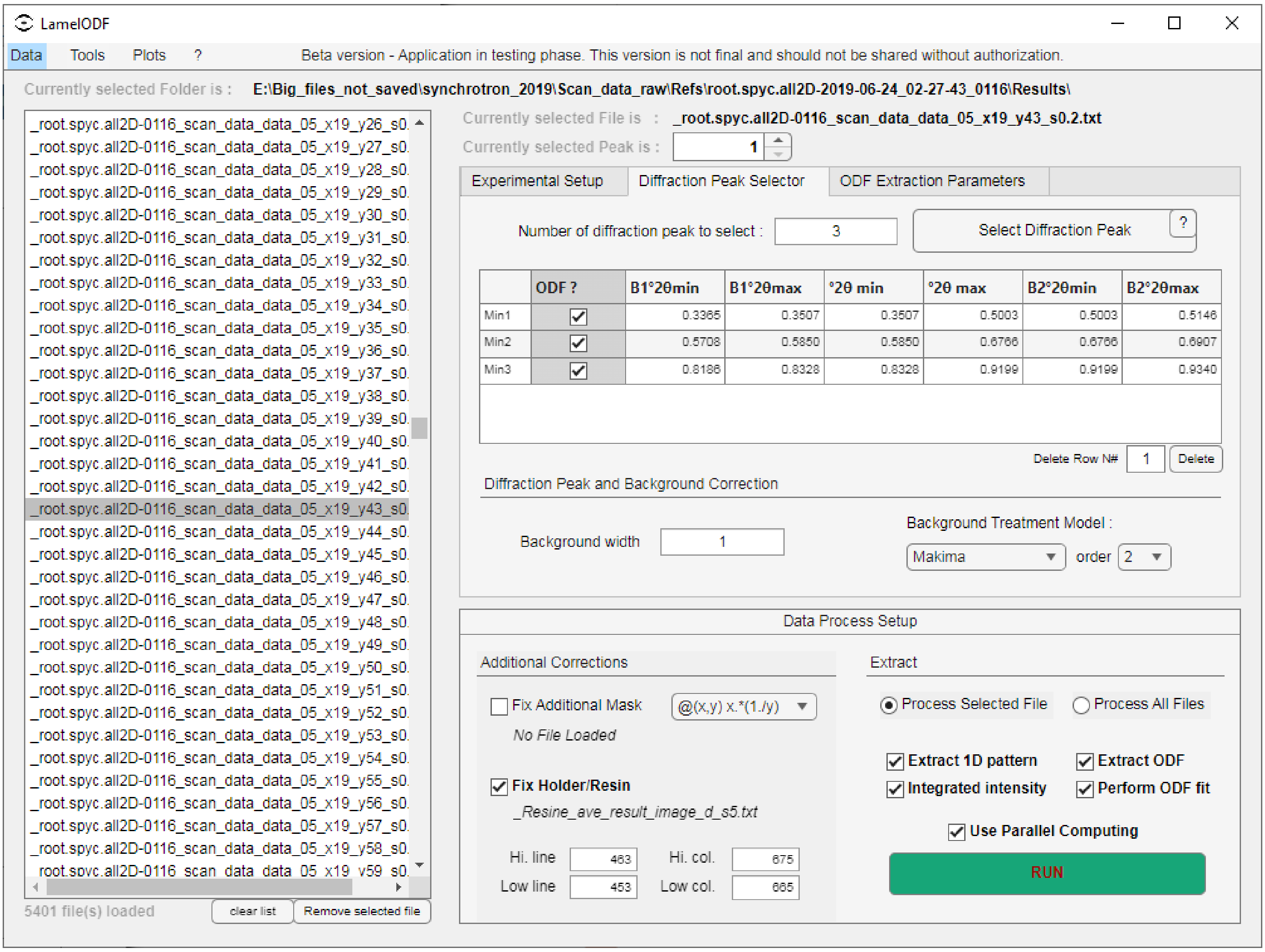
LamelODF
Data
Tools
Plots
?
Beta version - Application in testing phase. This version is not final and should not be shared without authorization.
Currently selected Folder is :
E:\Big_files_not_saved\synchrotron_2019\Scan_data_raw\Refs\root.spyc.all2D-2019-06-24_02-27-43_0116\Results\
Currently selected File is :
_root.spyc.all2D-0116_scan_data_data_05_x19_y43_s0.2.txt
Currently selected Peak is :
1
Experimental Setup
Diffraction Peak Selector
ODF Extraction Parameters
Number of diffraction peak to select :
3
Select Diffraction Peak
ODF ?
B1°2θmin
B1°2θmax
°2θ min
°2θ max
B2°2θmin
B2°2θmax
Min1 0.3365 0.3507 0.3507 0.5003 0.5003 0.5146
Min2 0.5708 0.5850 0.5850 0.6766 0.6766 0.6907
Min3 0.8186 0.8328 0.8328 0.9199 0.9199 0.9340
Delete Row N#
Delete
Diffraction Peak and Background Correction
Background width
Background Treatment Model :
Makima
order
Data Process Setup
Additional Corrections
Fix Additional Mask
@(x,y) x.*(1./y)
No File Loaded
Fix Holder/Resin
_Resine_ave_result_image_d_s5.txt
Hi. line
463
Hi. col.
675
Low line
453
Low col.
665
Extract
Process Selected File
Process All Files
Extract 1D pattern
Extract ODF
Integrated intensity
Perform ODF fit
Use Parallel Computing
RUN
5401 file(s) loaded
clear list
Remove selected file

## SI.1 C.

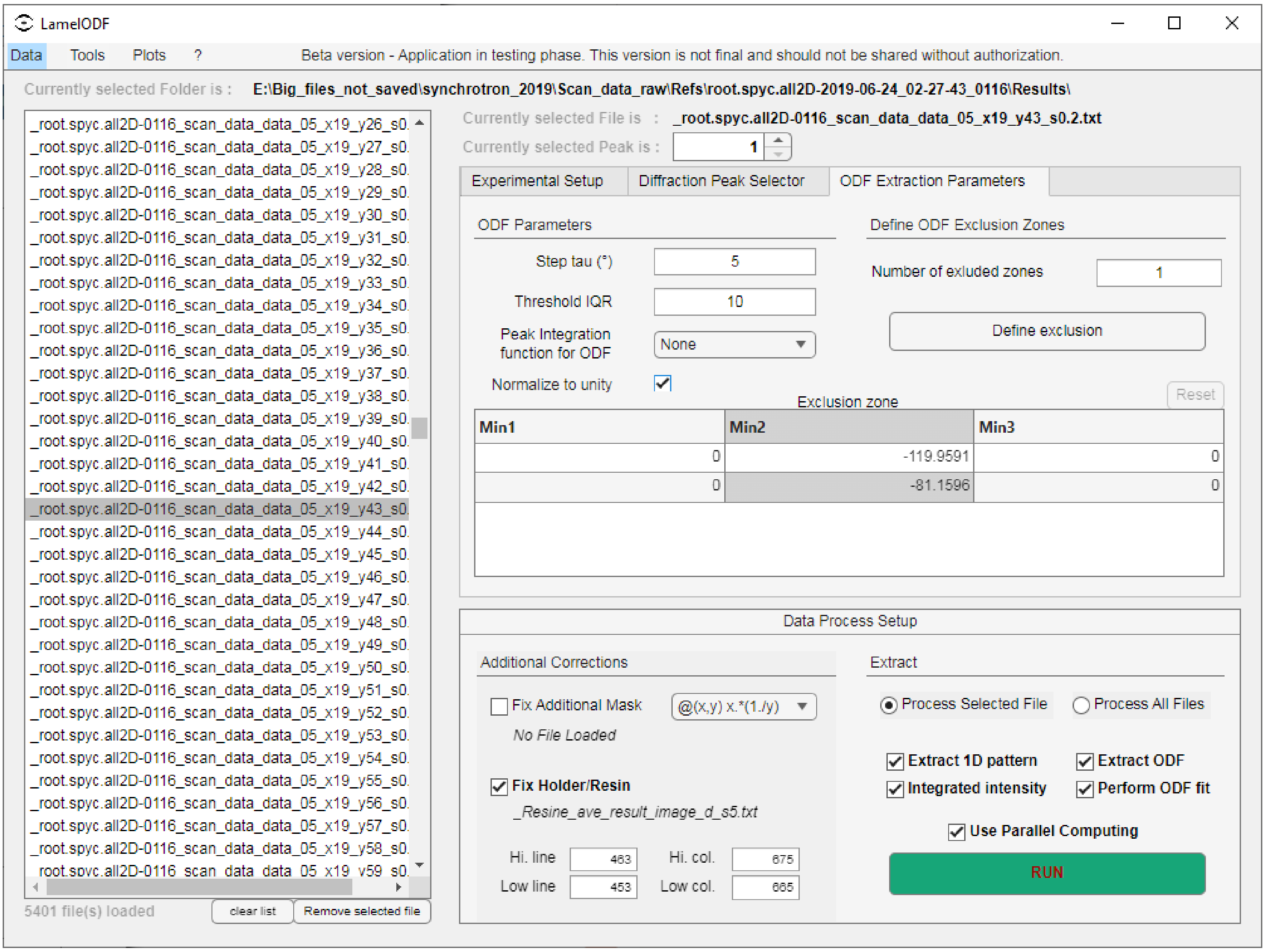
LamelODF
Data Tools Plots ?
Beta version - Application in testing phase. This version is not final and should not be shared without authorization.
Currently selected Folder is : E:\Big_files_not_saved\synchrotron_2019\Scan_data_raw\Refs\root.spyc.all2D-2019-06-24_02-27-43_0116\Results\
Currently selected File is : _root.spyc.all2D-0116_scan_data_data_05_x19_y43_s0.2.txt
Currently selected Peak is : 1
Experimental Setup
Diffraction Peak Selector
ODF Extraction Parameters
ODF Parameters
Step tau (°) 5
Threshold IQR 10
Peak Integration function for ODF None
Normalize to unity
Define ODF Exclusion Zones
Number of exluded zones 1
Define exclusion
Reset
Exclusion zone
Min1 Min2 Min3
0 -119.9591 0
0 -81.1596 0
Data Process Setup
Additional Corrections
Fix Additional Mask @(x,y) x.*(1./y)
No File Loaded
Fix Holder/Resin
_Resine_ave_result_image_d_s5.txt
Hi. line 463 Hi. col. 675
Low line 453 Low col. 665
Extract
Process Selected File
Process All Files
Extract 1D pattern
Extract ODF
Integrated intensity
Perform ODF fit
Use Parallel Computing
RUN
5401 file(s) loaded
clear list
Remove selected file

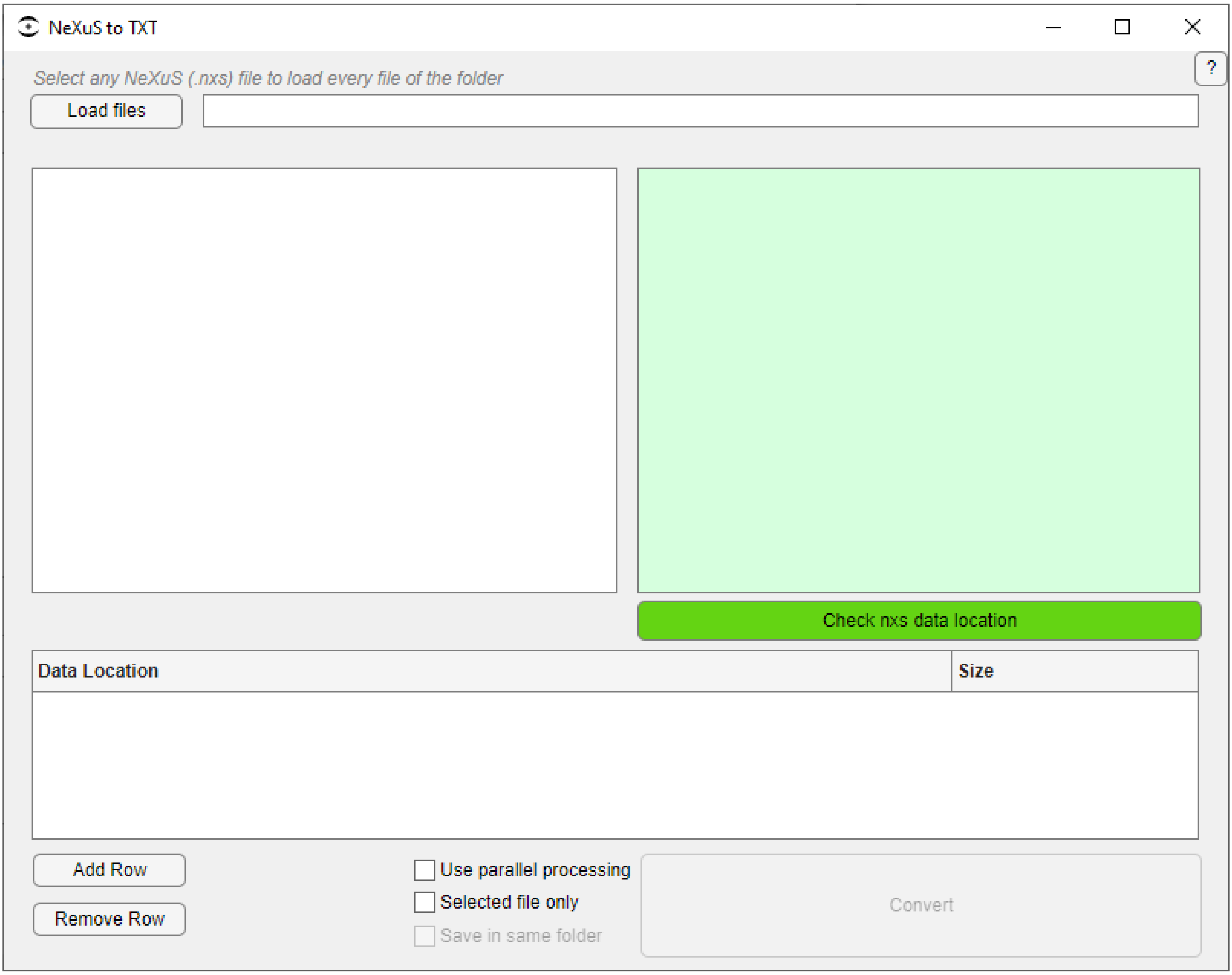


**SI.2:** LamelODF tool for direct conversion of NeXus files to .txt format. Options are available to locate various data stored within the file structure and output any required information as a .txt matrix file.

**SI.3 A.**

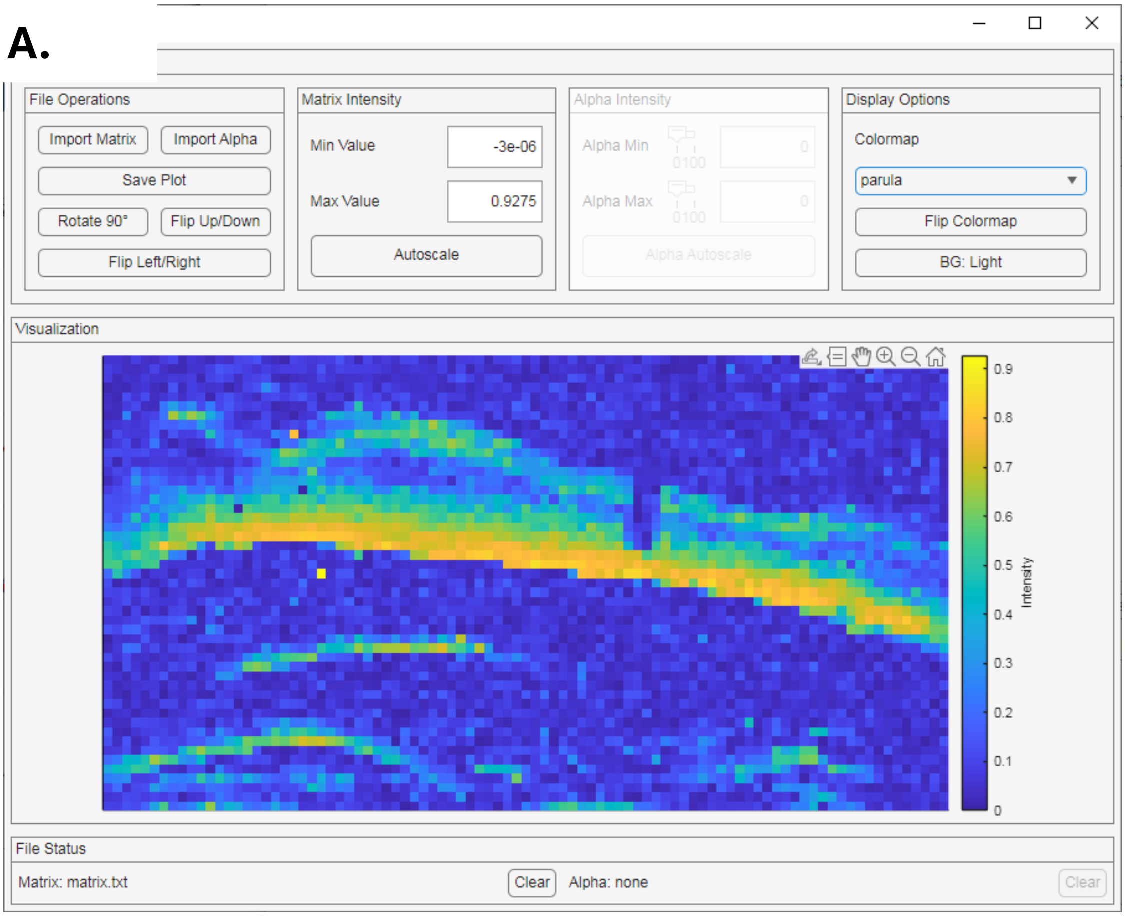


**SI.3 B.**

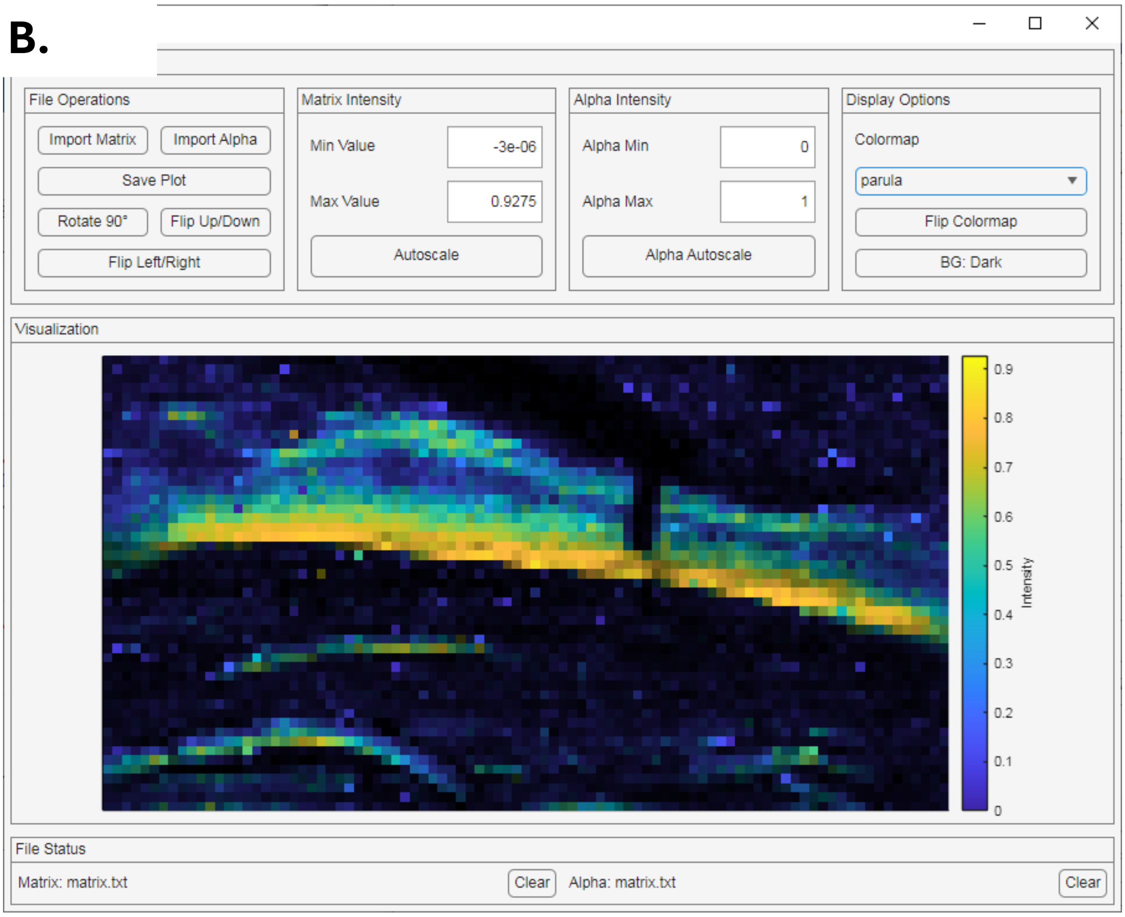


**SI.3:** A. Matrix viewer window tool, used to display various matrix-shaped results; here, an example of the P2 map for kaolinite in the Versailles topsoil sample. B. Similar to A, with the addition of an alpha mask (here, the intensity matrix of kaolinite for the same sample (SI. 4.)).

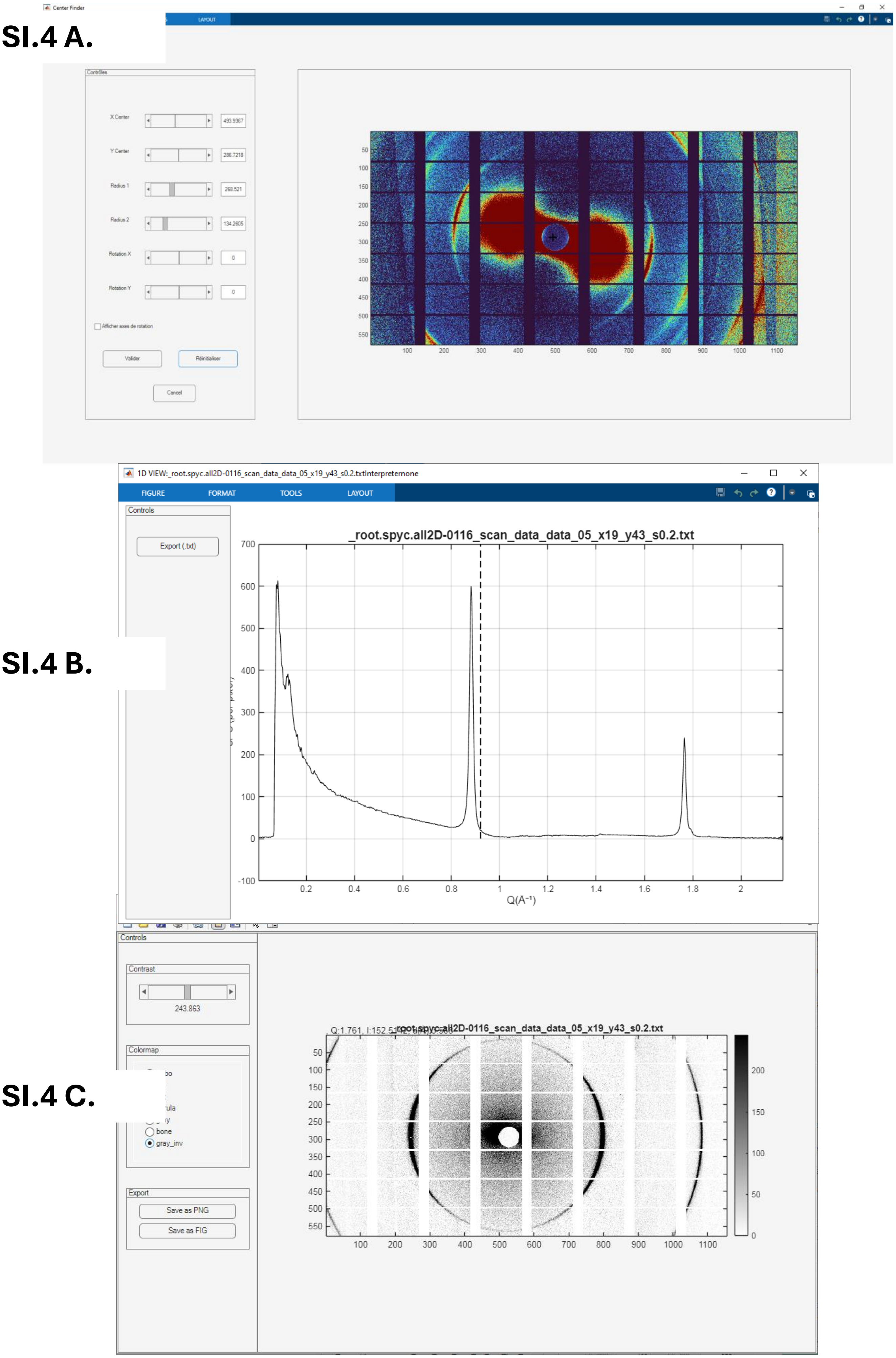


**SI.4:** A. Center finder tool for precise alignment of the beam center and tilt correction. Once aligned, and with proper experimental setup parameters entered, the 1D diffraction pattern in B can be computed through radial integration. C. Direct plot of the raw 2D XRD file data.

**SI.5 A.**

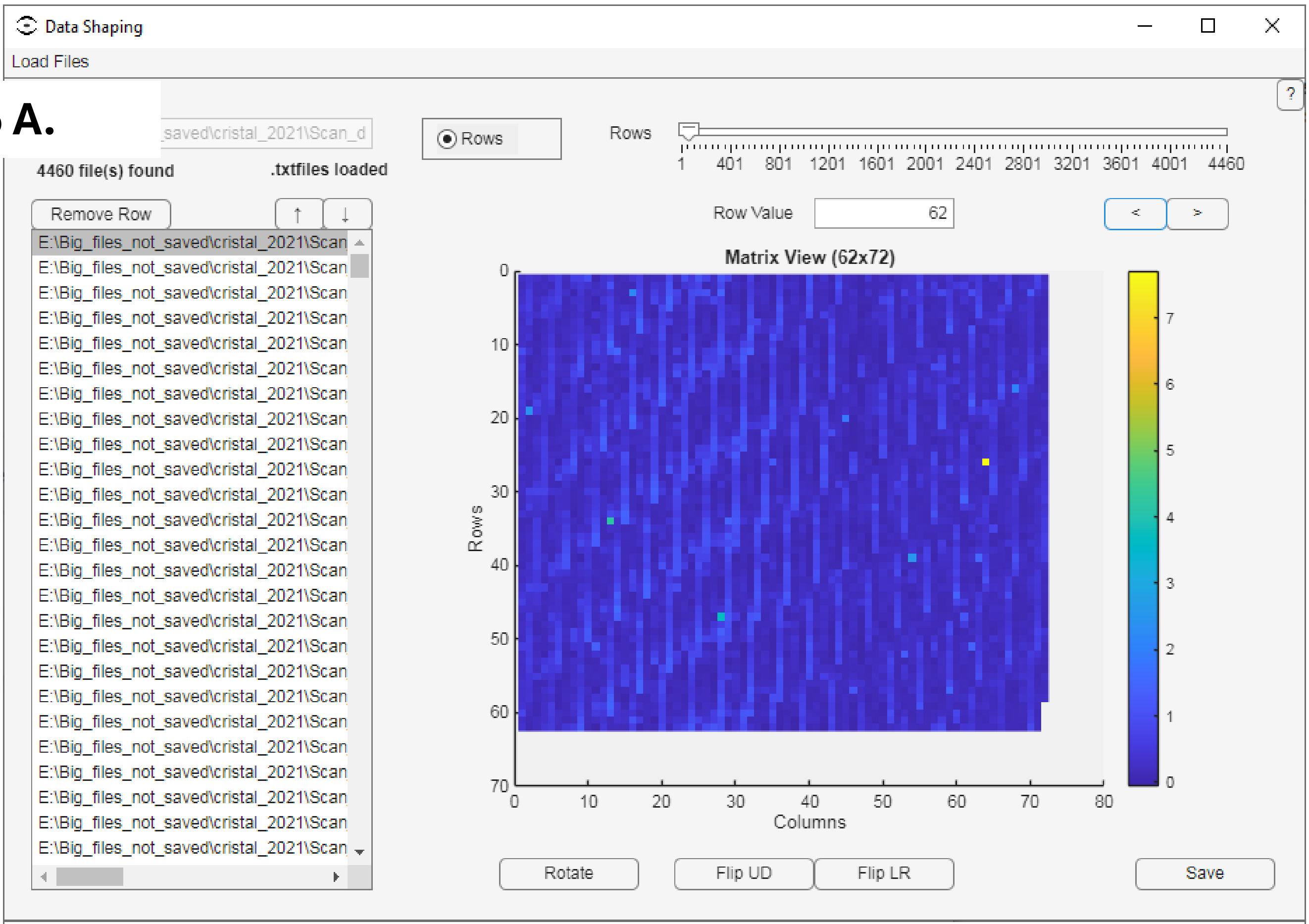


**SI.5 B.**

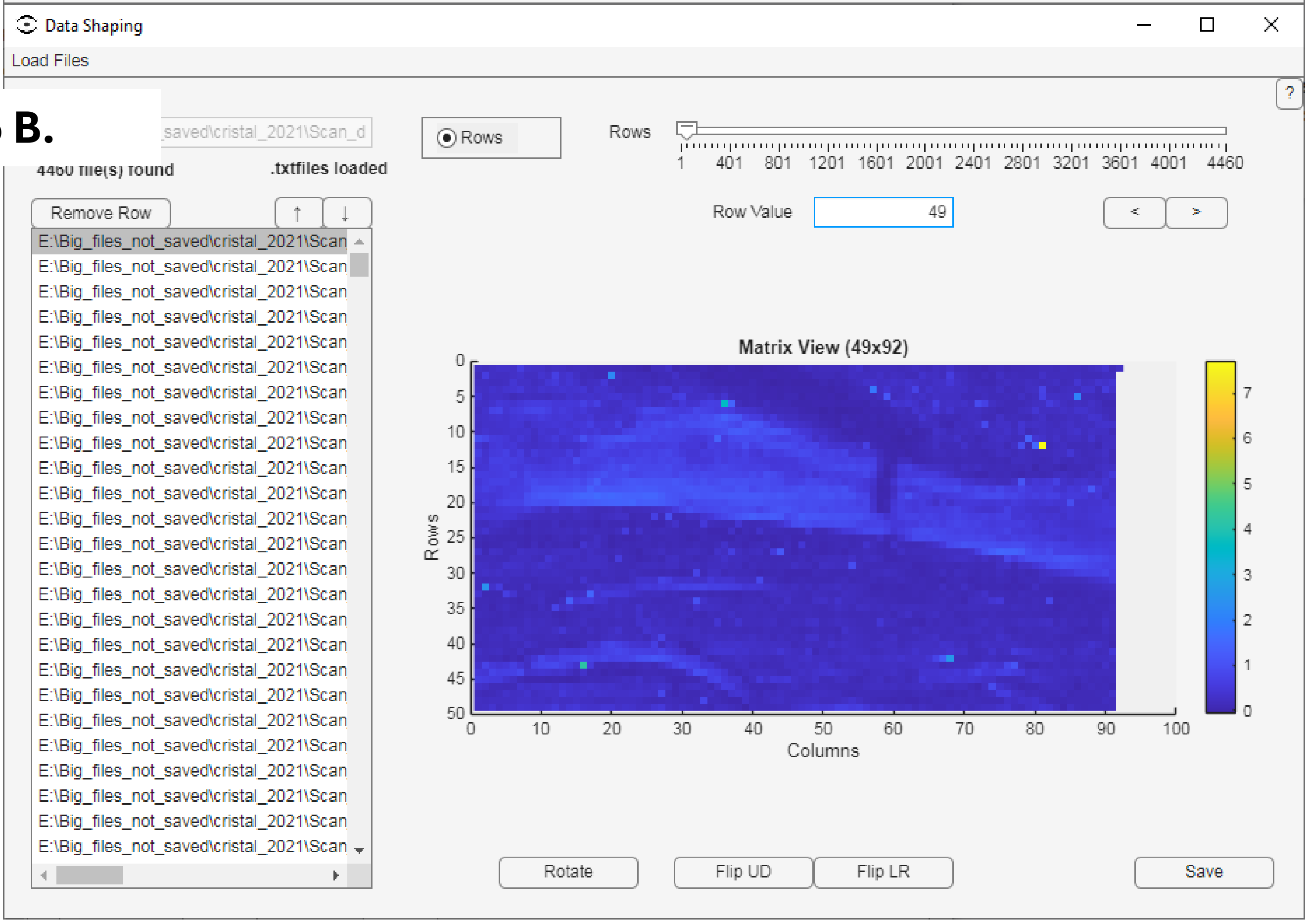


**SI.5:** Data shaping tool for loading individual files containing single values to be combined into a single matrix file once saved (for plotting as in SI.1). The process can be finely tuned line by line. A. Improperly shaped dataset. B. Correctly shaped dataset (number of rows vs. number of lines) for the kaolinite intensity parameter.

Analyzed pixel : Topsoil sample : X=44/Y=104

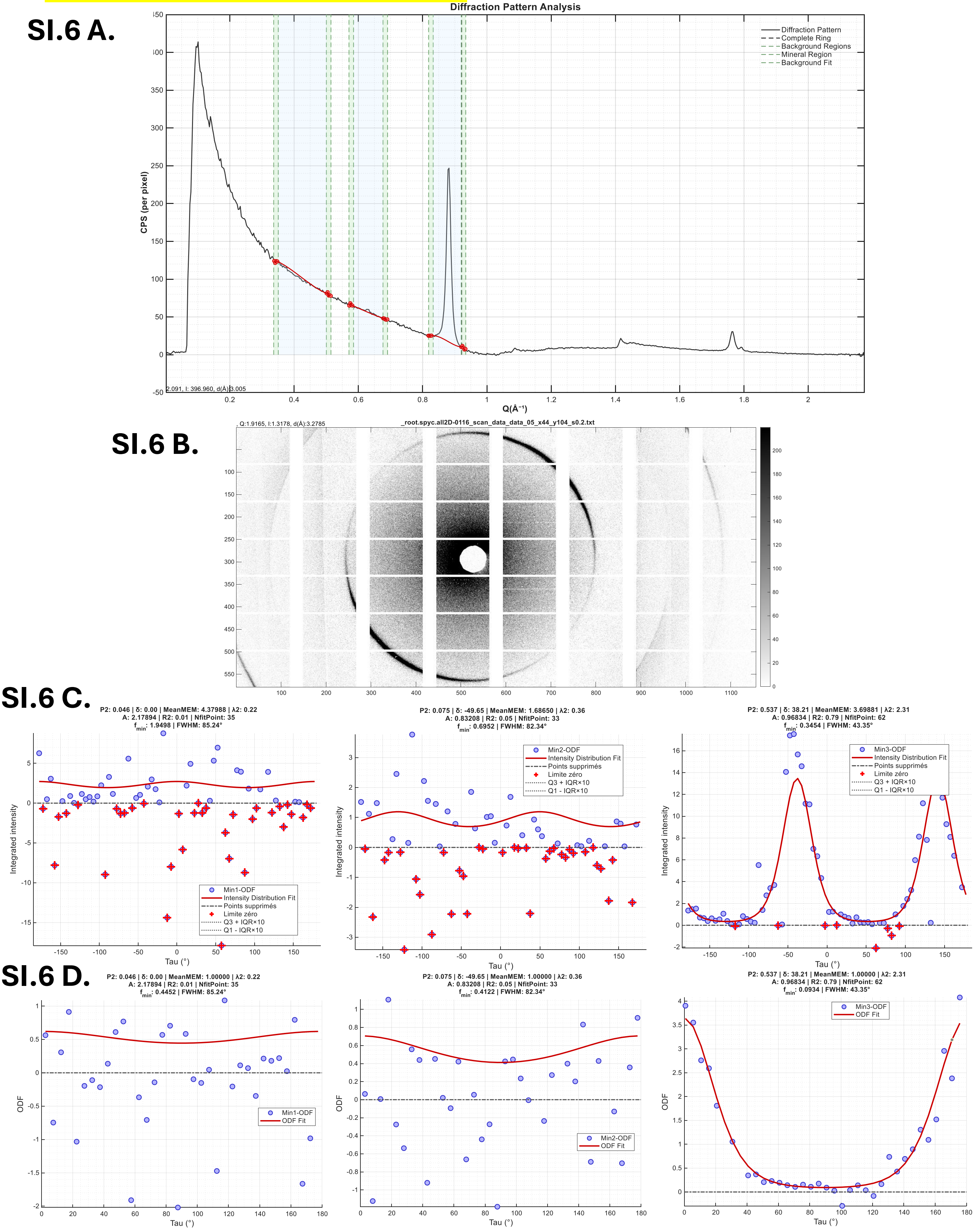


**SI.6:** Example of an analyzed 2D XRD point (X=44, Y=104) from the laboratory made clay sample. A. 1D integrated XRD pattern showing the selection of three minerals of interest, with selection boundaries in light green and background anchoring boundaries as dashed lines. The red curve represents the background model used for subtraction. B. Direct plot of raw 2D XRD data using the viewer tool (see SI.4). C and D. Azimuthal integrations corresponding to the boundaries selected in A (for min1, min2, and min3). C. Direct integrated intensity with the MEM fit. D. Normalized and symmetrized ODF for the same minerals. Various fitting parameters are indicated on the plots.

Analyzed pixel : Topsoil sample : X=15/Y=82

SI.7 A.

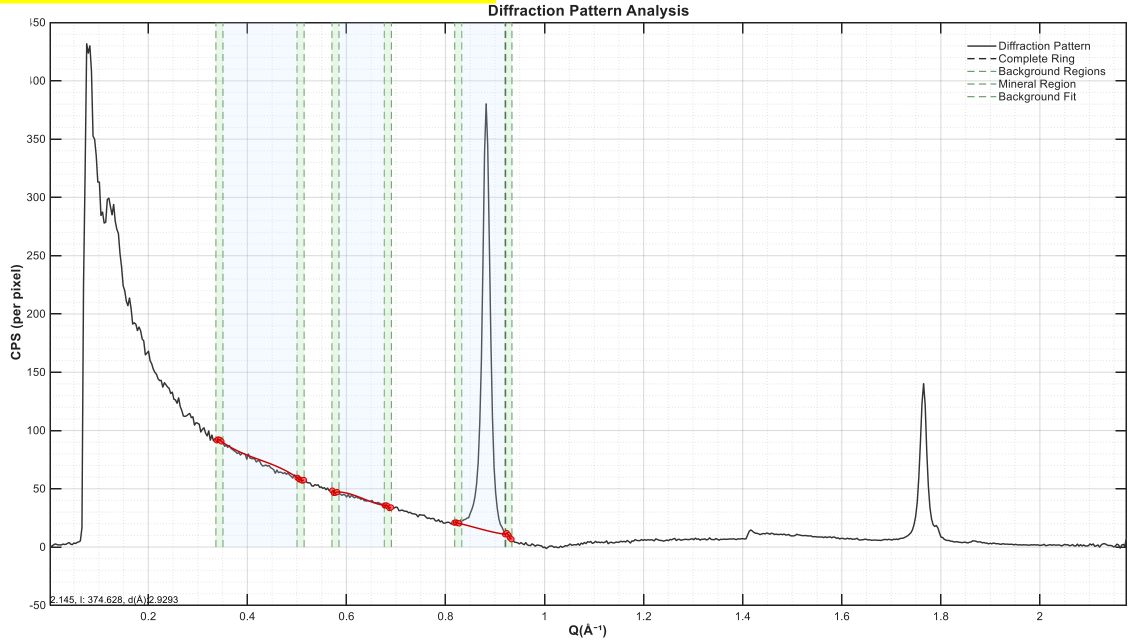


SI.7 B.

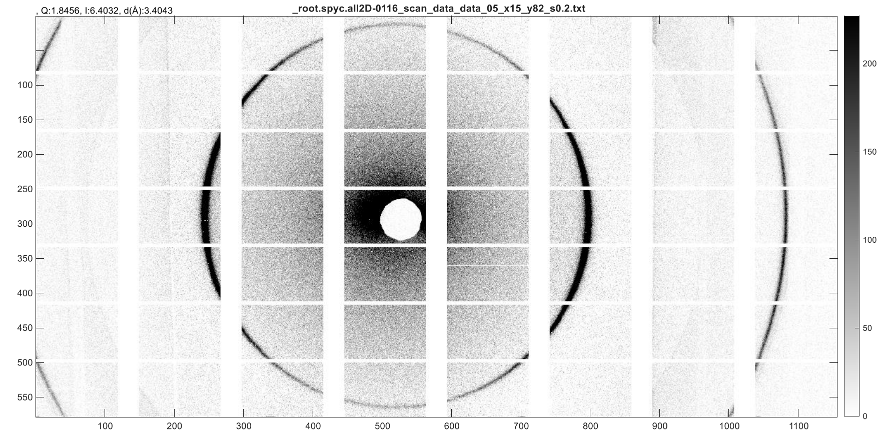


SI.7 C.

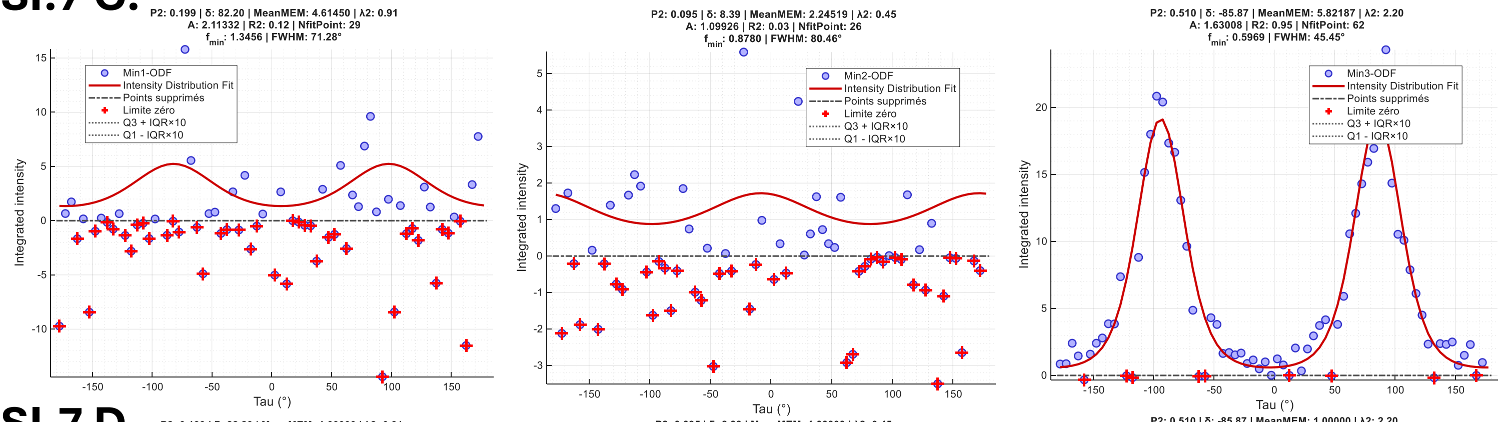


SI.7 D.

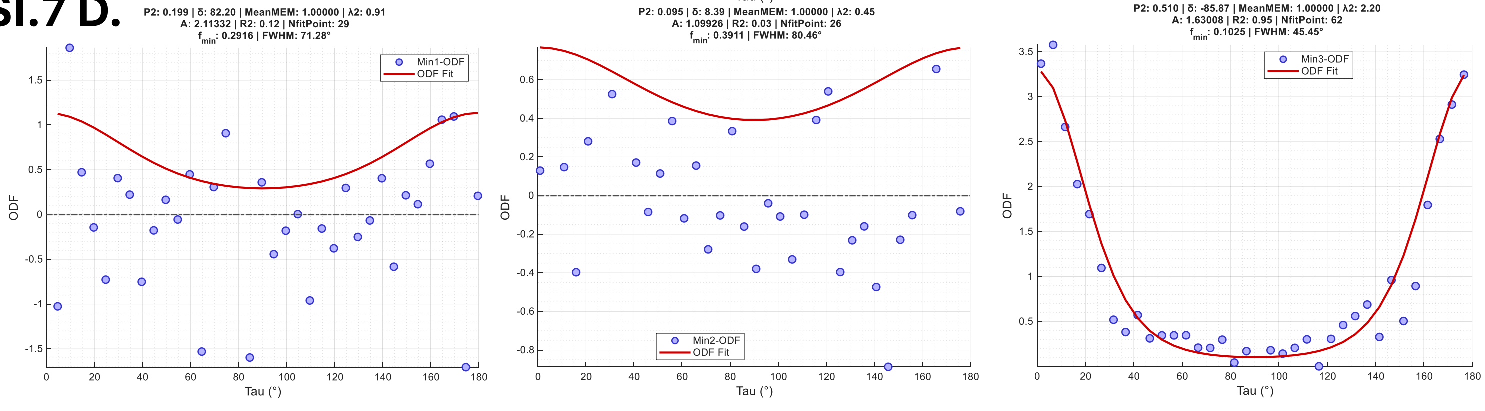


**SI.7:** Example of an analyzed 2D XRD point (X=15, Y=82) from the laboratory made clay sample. A. 1D integrated XRD pattern showing the selection of three minerals of interest, with selection boundaries in light green and background anchoring boundaries as dashed lines. The red curve represents the background model used for subtraction. B. Direct plot of raw 2D XRD data using the viewer tool (see SI.4). C and D. Azimuthal integrations corresponding to the boundaries selected in A (for min1, min2, and min3). C. Direct integrated intensity with the MEM fit. D. Normalized and symmetrized ODF for the same minerals. Various fitting parameters are indicated on the plots.

Analyzed pixel : Topsoil sample : X=27/Y=57

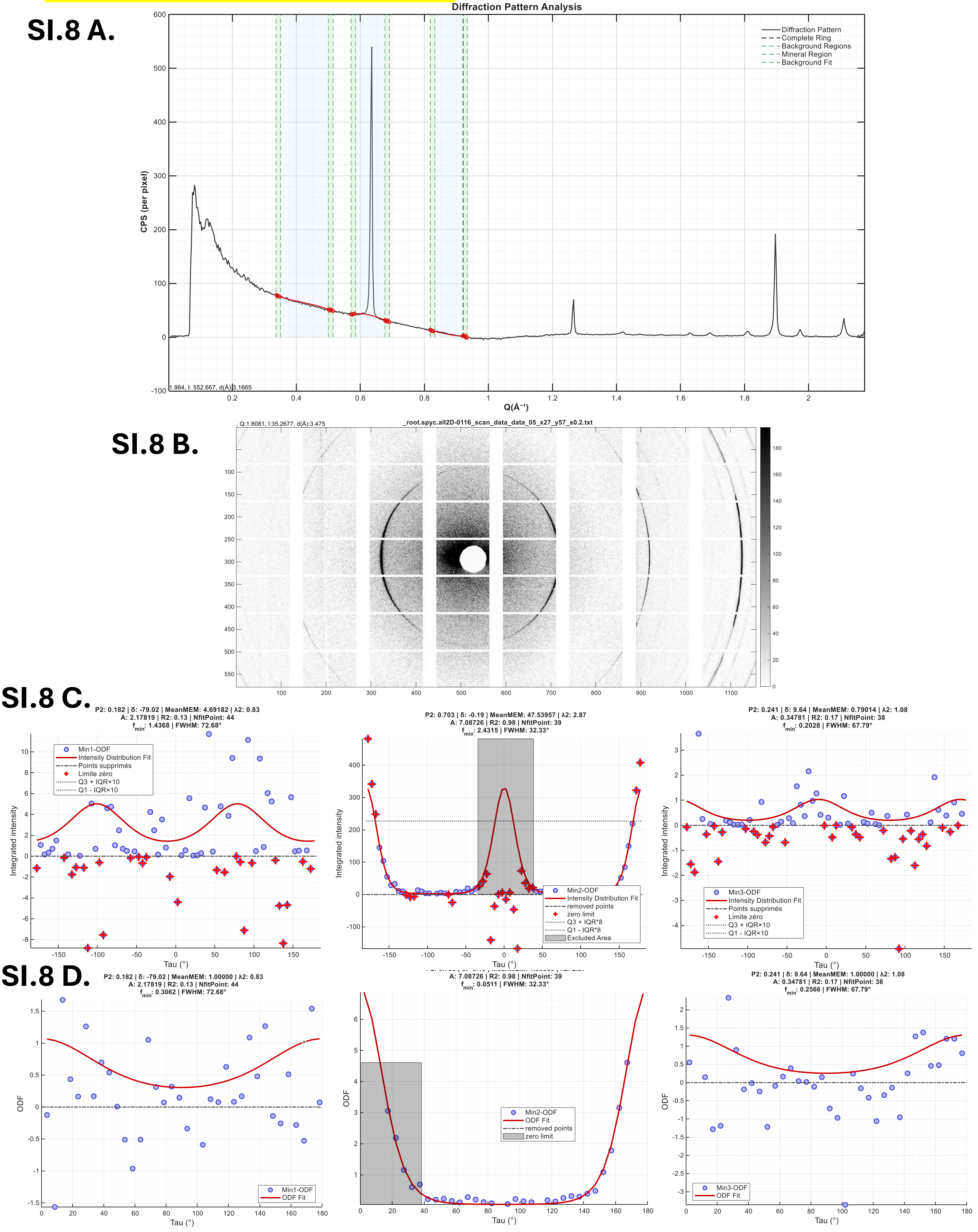


**SI.8:** Example of an analyzed 2D XRD point (X=27, Y=57) from the laboratory made clay sample. A. 1D integrated XRD pattern showing the selection of three minerals of interest, with selection boundaries in light green and background anchoring boundaries as dashed lines. The red curve represents the background model used for subtraction. B. Direct plot of raw 2D XRD data using the viewer tool (see SI.4). C and D. Azimuthal integrations corresponding to the boundaries selected in A (for min1, min2, and min3). C. Direct integrated intensity with the MEM fit. D. Normalized and symmetrized ODF for the same minerals. Various fitting parameters are indicated on the plots.

Analyzed pixel : Topsoil sample : X=21/Y=09

SI.9 A.

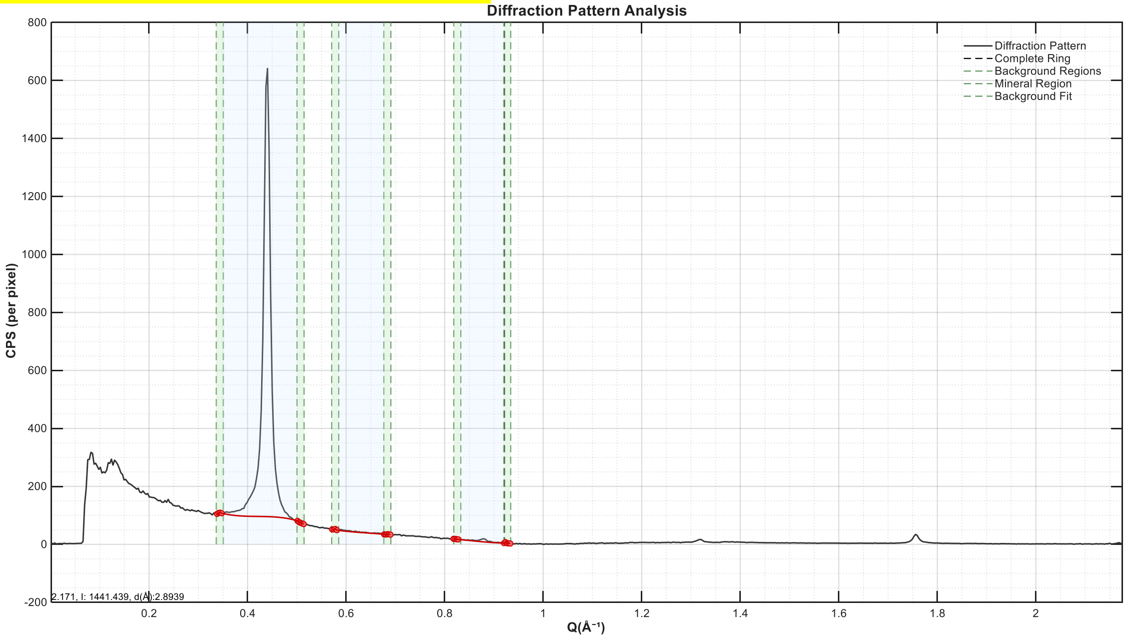


SI.9 B.

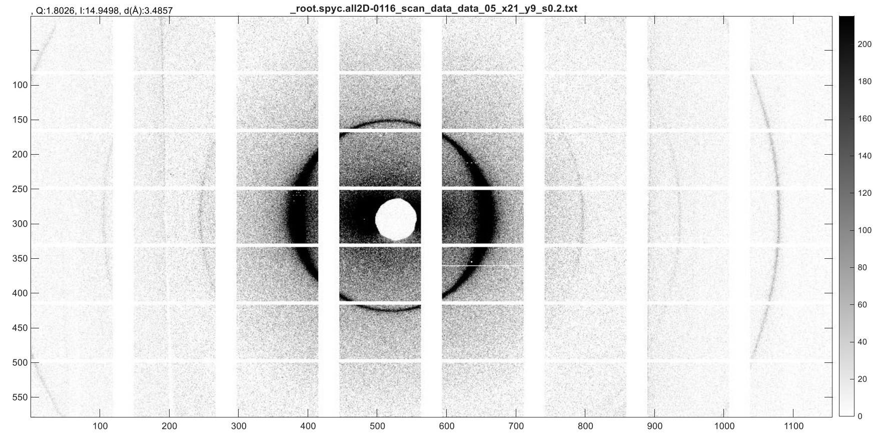


SI.9 C.

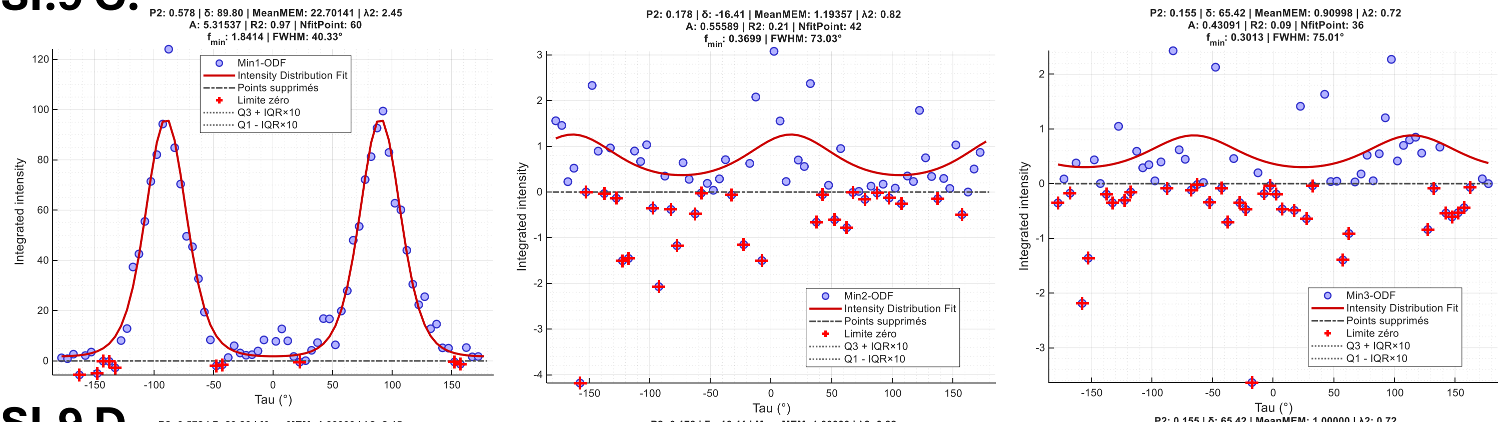


SI.9 D.

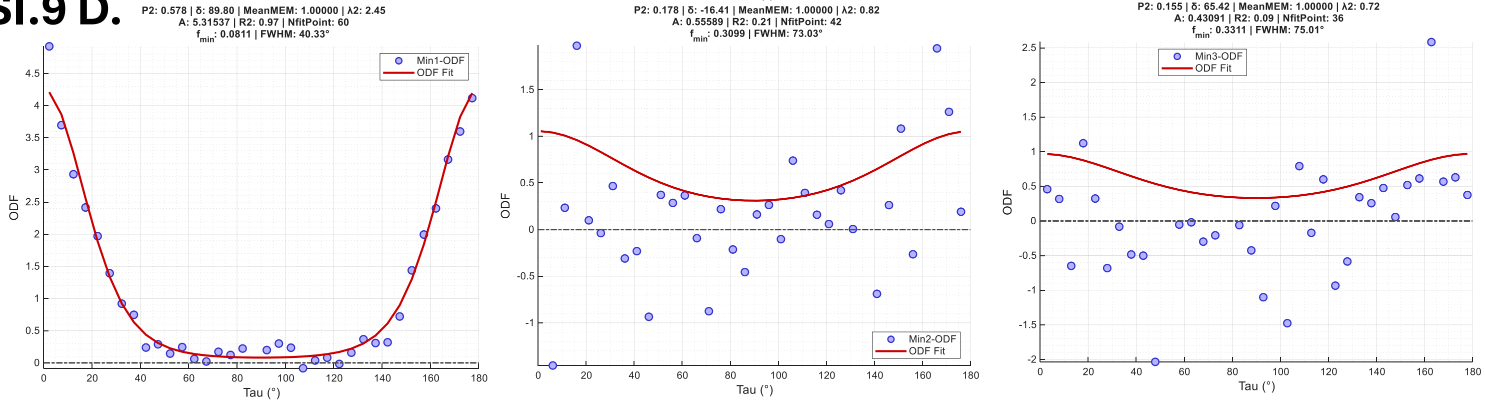


**SI.9:** Example of an analyzed 2D XRD point (X=21, Y=09) from the laboratory made clay sample. A. 1D integrated XRD pattern showing the selection of three minerals of interest, with selection boundaries in light green and background anchoring boundaries as dashed lines. The red curve represents the background model used for subtraction. B. Direct plot of raw 2D XRD data using the viewer tool (see SI.4). C and D. Azimuthal integrations corresponding to the boundaries selected in A (for min1, min2, and min3). C. Direct integrated intensity with the MEM fit. D. Normalized and symmetrized ODF for the same minerals. Various fitting parameters are indicated on the plots.

Analyzed pixel : Topsoil sample : X=19/Y=43

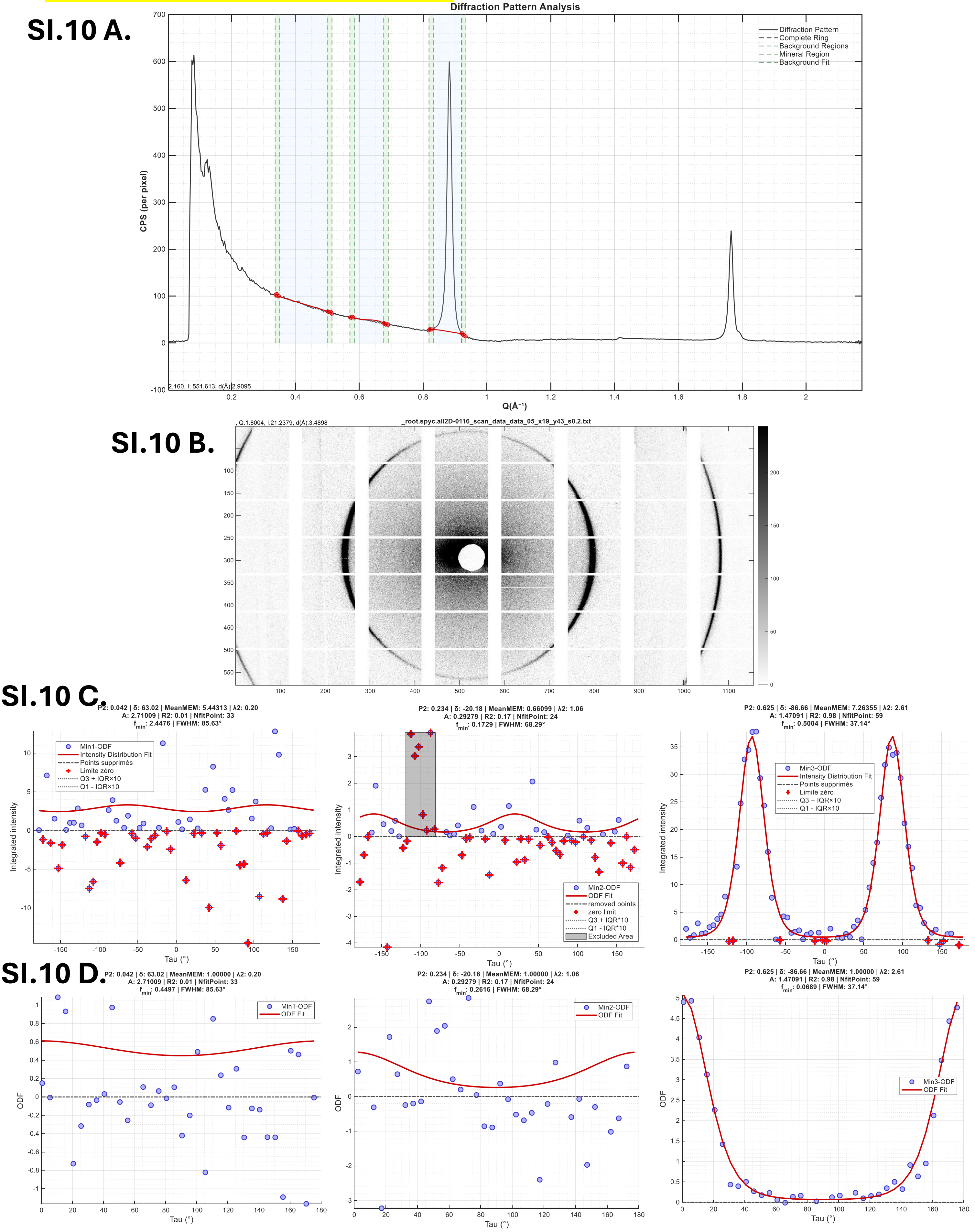


**SI.10**: Example of an analyzed 2D XRD point (X=19, Y=43) from the laboratory made clay sample. A. 1D integrated XRD pattern showing the selection of three minerals of interest, with selection boundaries in light green and background anchoring boundaries as dashed lines. The red curve represents the background model used for subtraction. B. Direct plot of raw 2D XRD data using the viewer tool (see SI.4). C and D. Azimuthal integrations corresponding to the boundaries selected in A (for min1, min2, and min3). C. Direct integrated intensity with the MEM fit. D. Normalized and symmetrized ODF for the same minerals. Various fitting parameters are indicated on the plots.

Analyzed pixel : Topsoil sample : X=20/Y=19

**SI.11 A.**

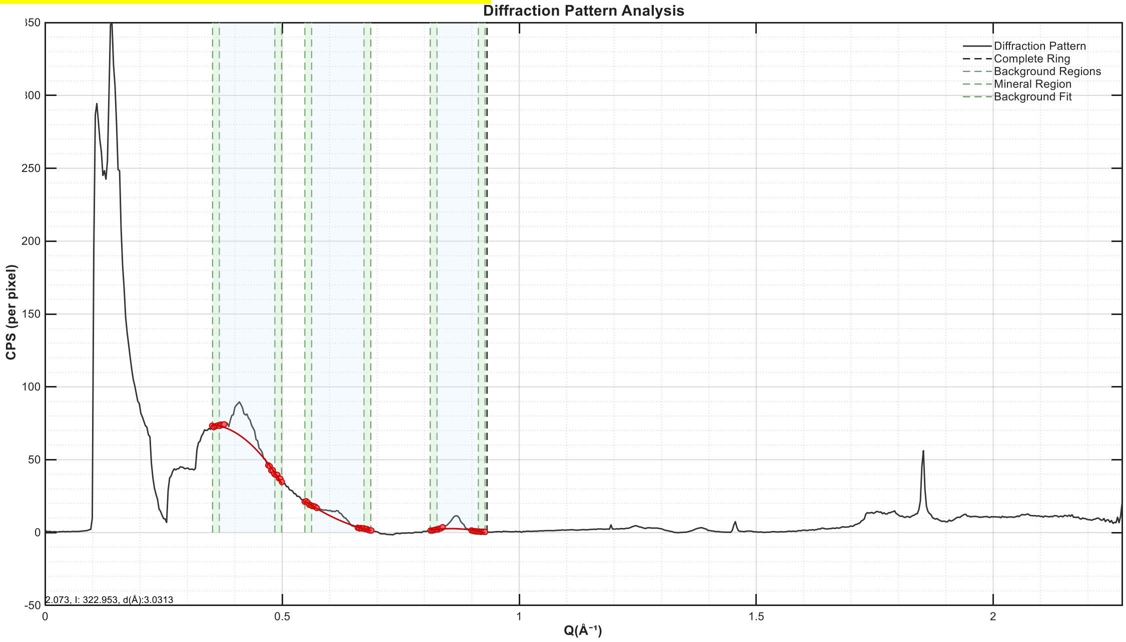


**SI.11 B.**

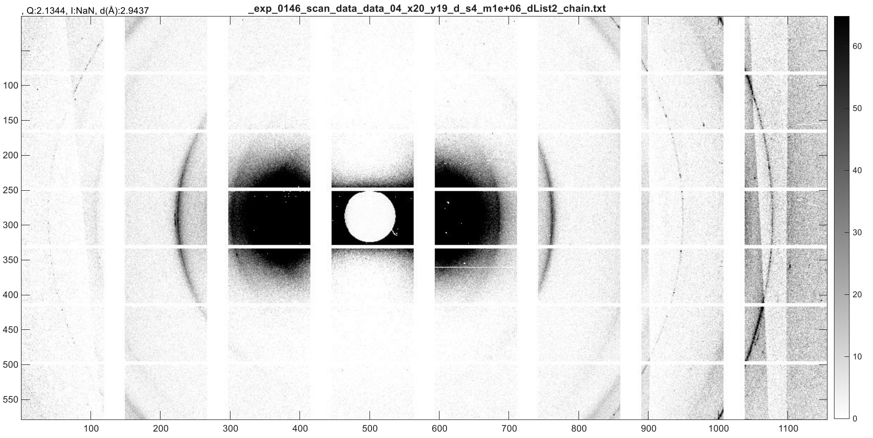


**SI.11 C.**

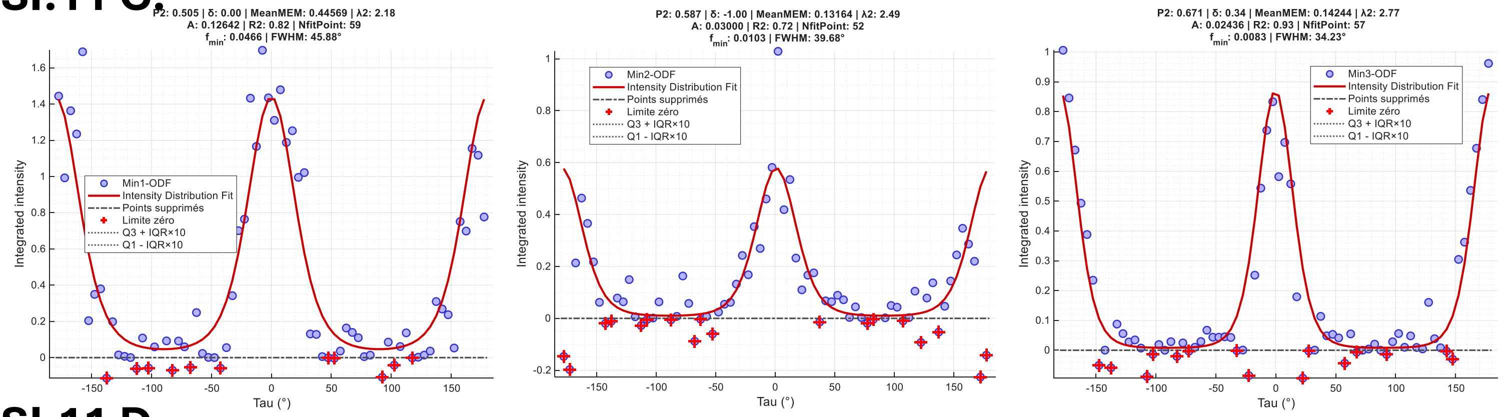


**SI.11 D.**

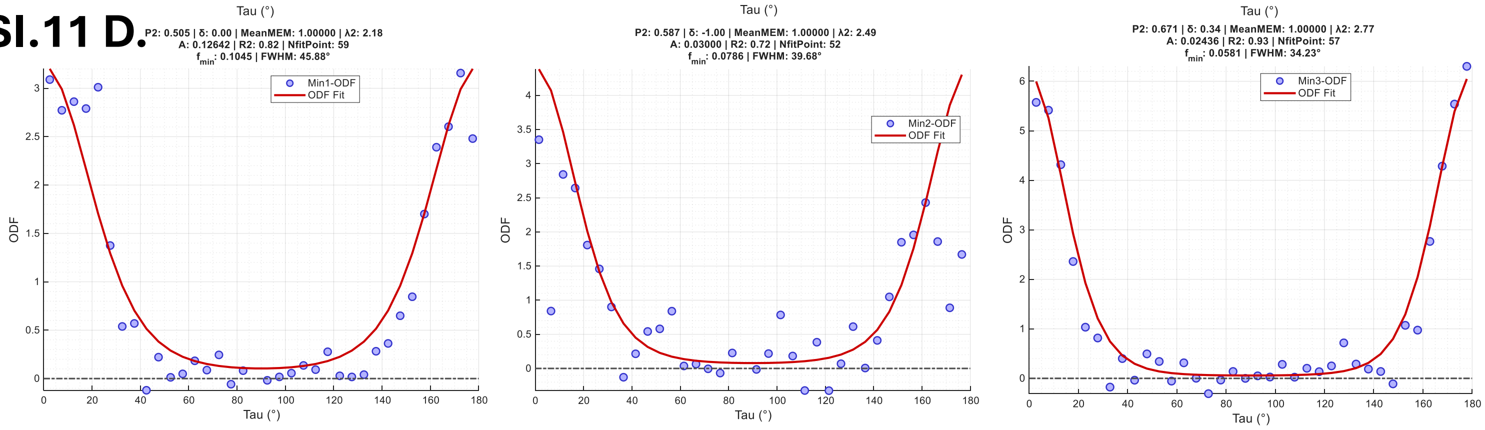


**SI.11:** Example of an analyzed 2D XRD point (X=20, Y=19) from the Versailles topsoil sample. A. 1D integrated XRD pattern showing the selection of three minerals of interest, with selection boundaries in light green and background anchoring boundaries as dashed lines. The red curve represents the background model used for subtraction. B. Direct plot of raw 2D XRD data using the viewer tool (see SI.4). C and D. Azimuthal integrations corresponding to the boundaries selected in A (for min1, min2, and min3). C. Direct integrated intensity with the MEM fit. D. Normalized and symmetrized ODF for the same minerals. Various fitting parameters are indicated on the plots.

Analyzed pixel : Topsoil sample : X=87/Y=29

SI.12 A.

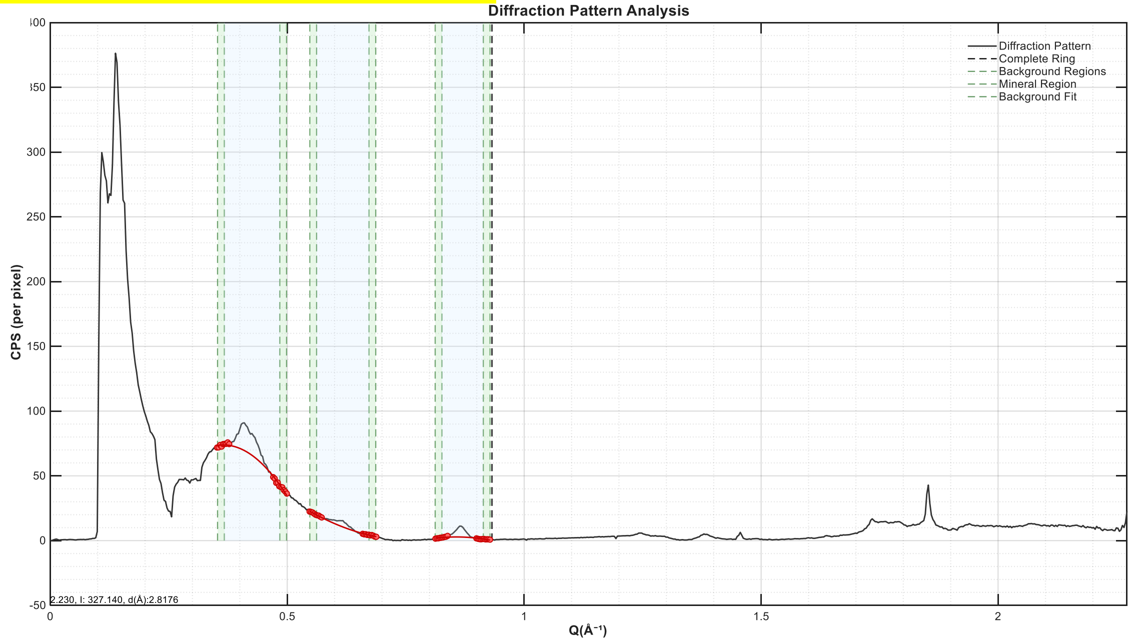


SI.12 B.

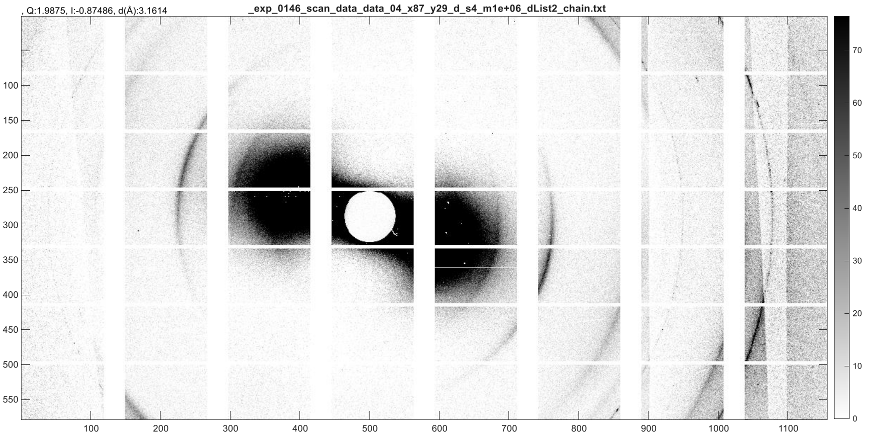


SI.12 C.

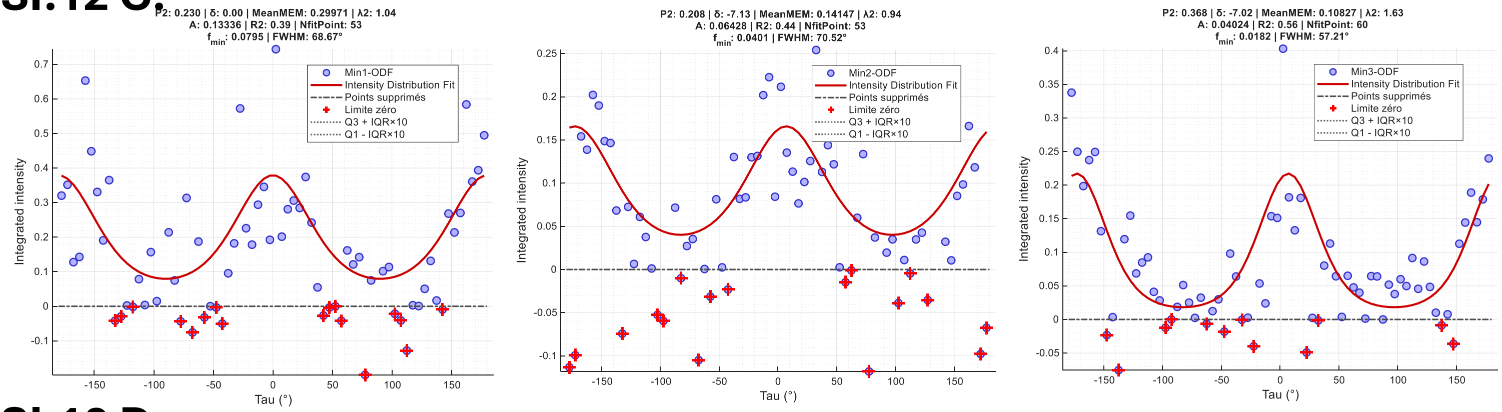


SI.12 D.

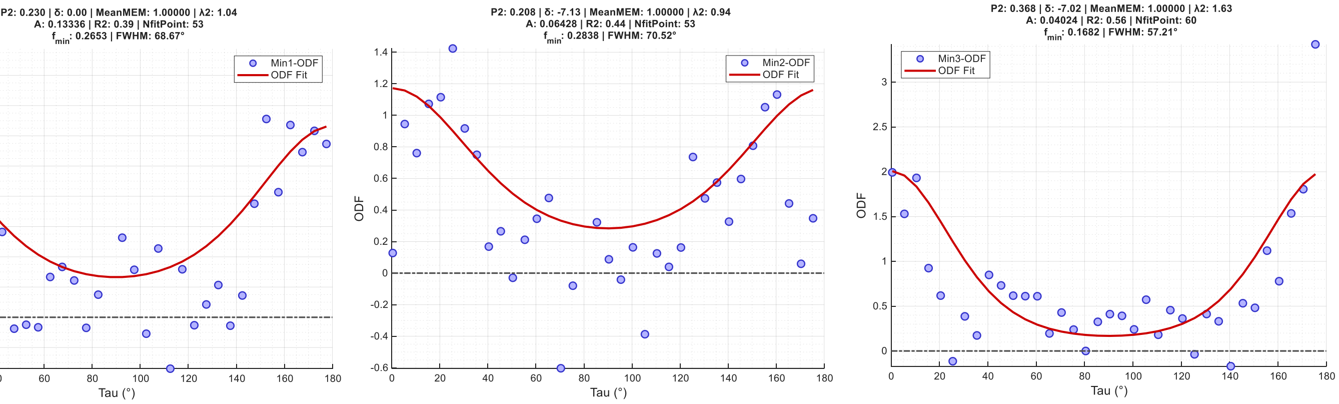


**SI.12:** Example of an analyzed 2D XRD point (X=87, Y=29) from the Versailles topsoil sample. A. 1D integrated XRD pattern showing the selection of three minerals of interest, with selection boundaries in light green and background anchoring boundaries as dashed lines. The red curve represents the background model used for subtraction. B. Direct plot of raw 2D XRD data using the viewer tool (see SI.4). C and D. Azimuthal integrations corresponding to the boundaries selected in A (for min1, min2, and min3). C. Direct integrated intensity with the MEM fit. D. Normalized and symmetrized ODF for the same minerals. Various fitting parameters are indicated on the plots.

Analyzed pixel : Topsoil sample : X=76/Y=44

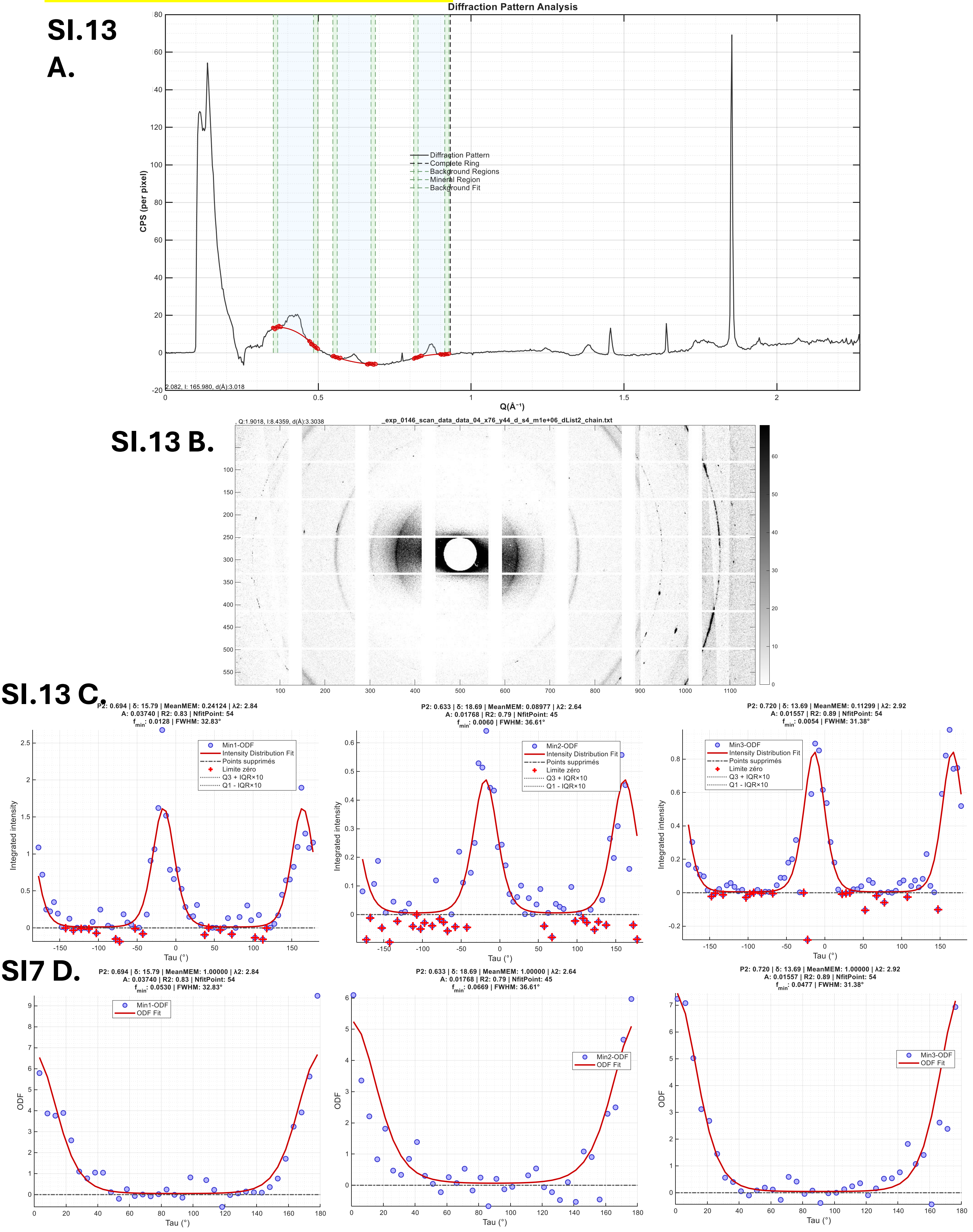


**SI.13:** Example of an analyzed 2D XRD point (X=76, Y=44) from the Versailles topsoil sample. A. 1D integrated XRD pattern showing the selection of three minerals of interest, with selection boundaries in light green and background anchoring boundaries as dashed lines. The red curve represents the background model used for subtraction. B. Direct plot of raw 2D XRD data using the viewer tool (see SI.4). C and D. Azimuthal integrations corresponding to the boundaries selected in A (for min1, min2, and min3). C. Direct integrated intensity with the MEM fit. D. Normalized and symmetrized ODF for the same minerals. Various fitting parameters are indicated on the plots.

Analyzed pixel : Topsoil sample : X=06/Y=18

**SI.14 A.**

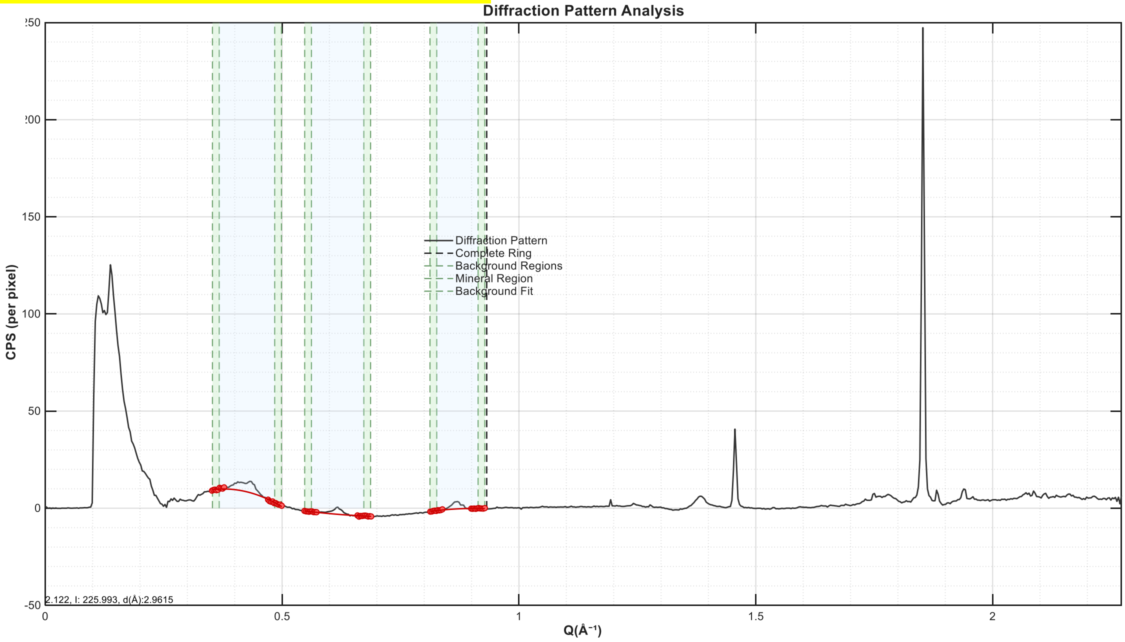


**SI.14 B.**

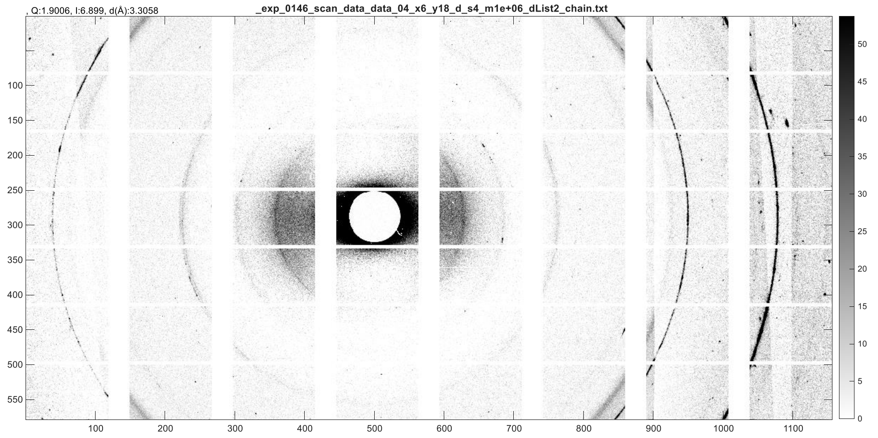


**SI.14 C.**

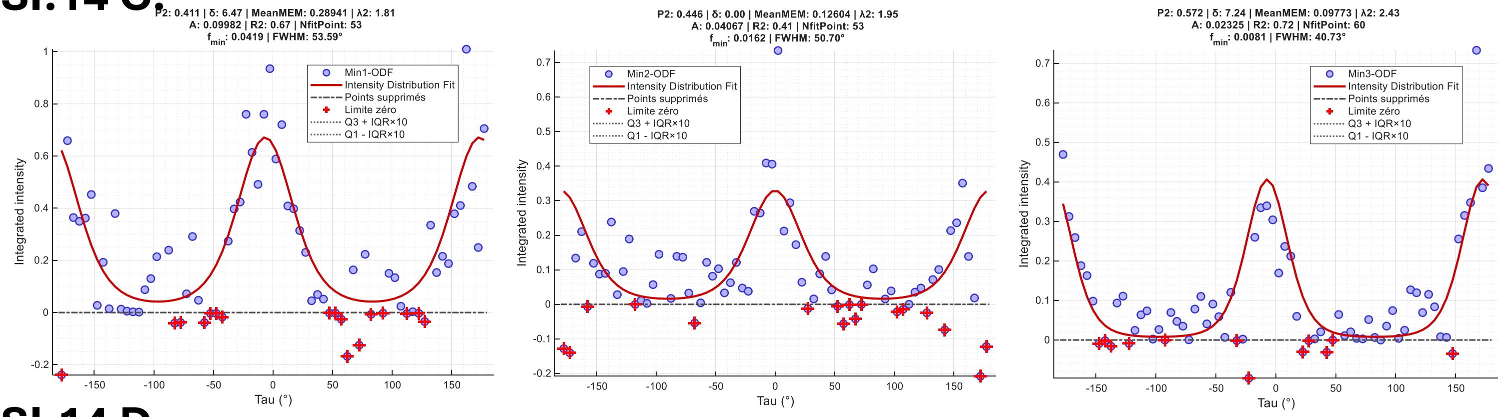


**SI.14 D.**

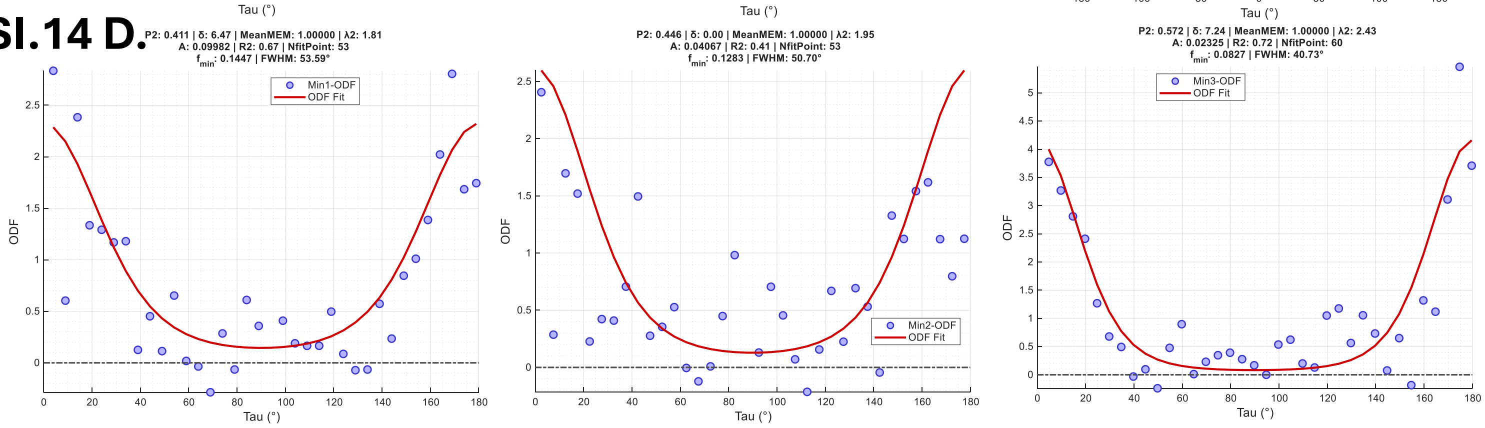


**SI.14:** Example of an analyzed 2D XRD point (X=06, Y=18) from the Versailles topsoil sample. A. 1D integrated XRD pattern showing the selection of three minerals of interest, with selection boundaries in light green and background anchoring boundaries as dashed lines. The red curve represents the background model used for subtraction. B. Direct plot of raw 2D XRD data using the viewer tool (see SI.4). C and D. Azimuthal integrations corresponding to the boundaries selected in A (for min1, min2, and min3). C. Direct integrated intensity with the MEM fit. D. Normalized and symmetrized ODF for the same minerals. Various fitting parameters are indicated on the plots.

Analyzed pixel : Topsoil sample : X=59/Y=34

# SI.15 A.

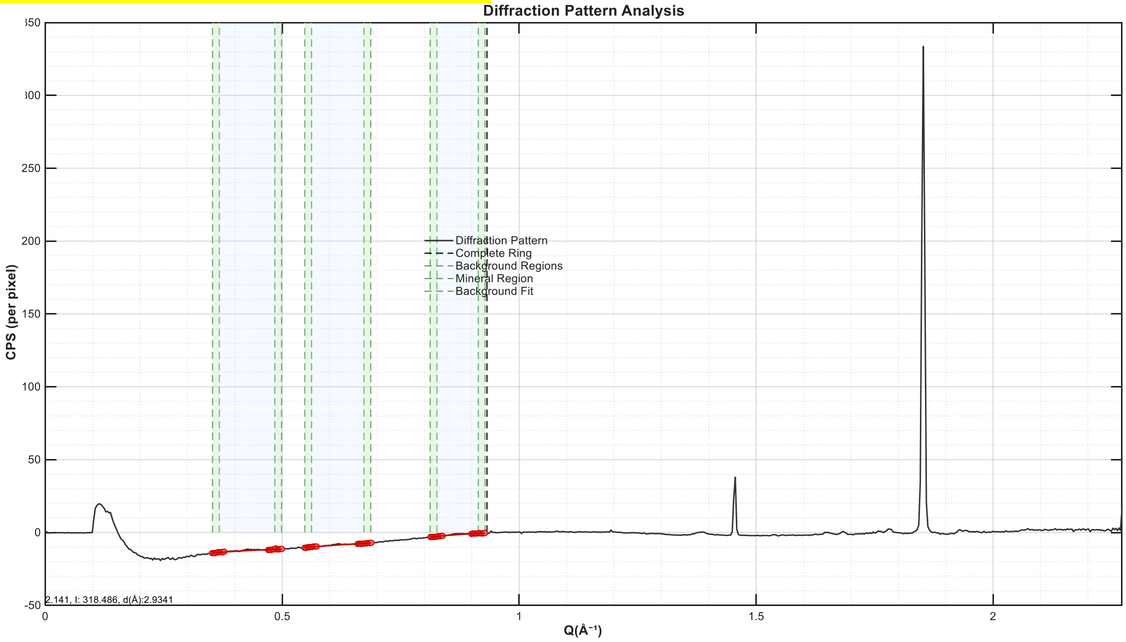


# SI.15 B.

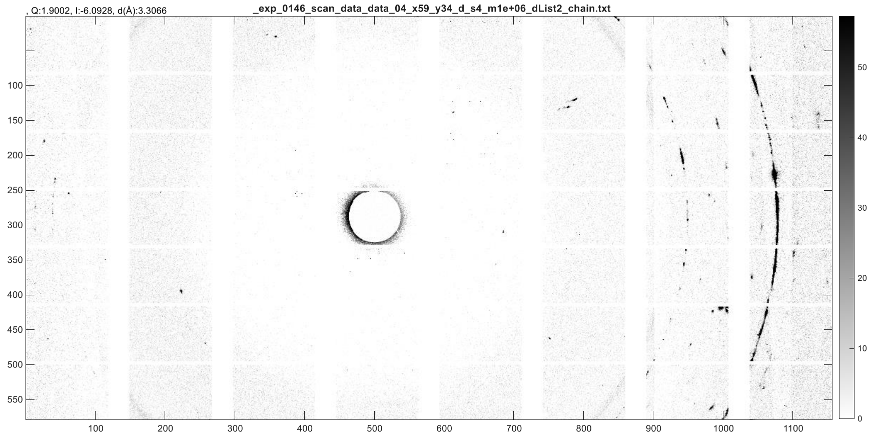


# SI.15 C.

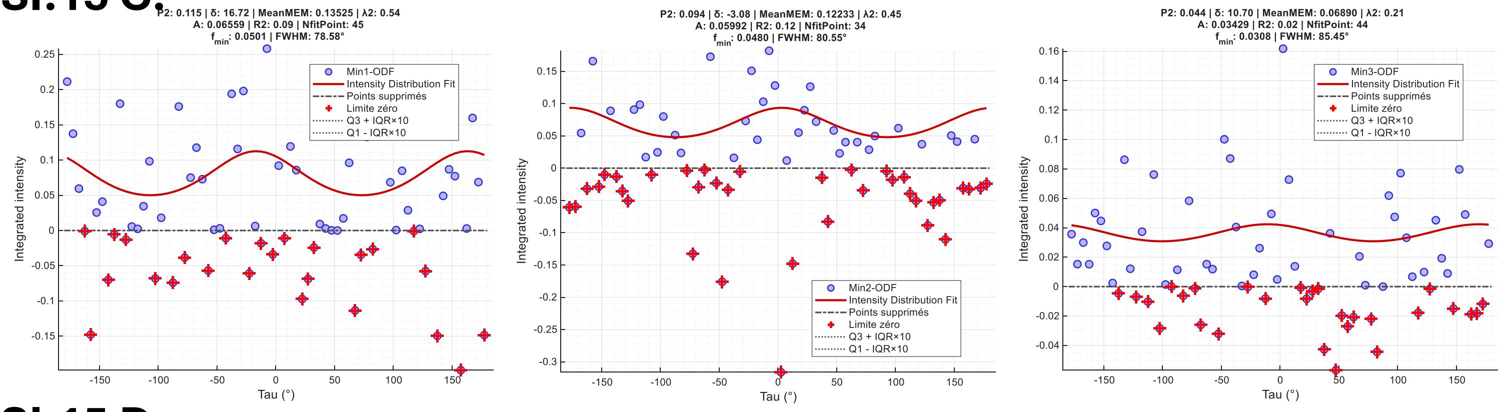


# SI.15 D.

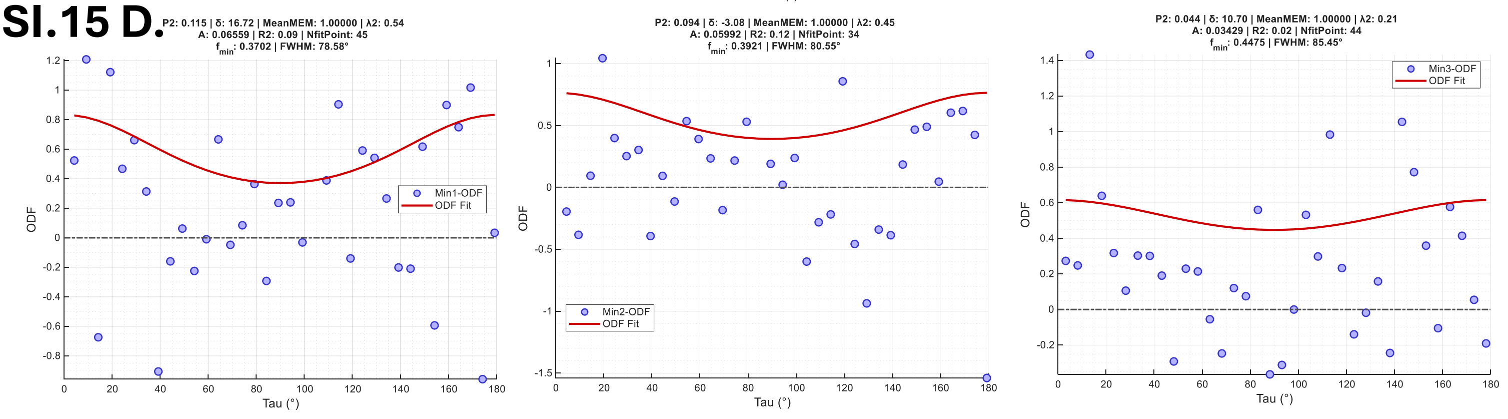


**SI.15:** Example of an analyzed 2D XRD point (X=59, Y=34) from the Versailles topsoil sample. A. 1D integrated XRD pattern showing the selection of three minerals of interest, with selection boundaries in light green and background anchoring boundaries as dashed lines. The red curve represents the background model used for subtraction. B. Direct plot of raw 2D XRD data using the viewer tool (see SI.4). C and D. Azimuthal integrations corresponding to the boundaries selected in A (for min1, min2, and min3). C. Direct integrated intensity with the MEM fit. D. Normalized and symmetrized ODF for the same minerals. Various fitting parameters are indicated on the plots.
